# Modulating the Charge Transfer Channels via Constructing Charge‐Assisted Hydrogen‐Bonded Organic Frameworks for Enhanced Photosynthesis of Hydrogen Peroxide

**DOI:** 10.1002/advs.75201

**Published:** 2026-04-07

**Authors:** Yajun Zhao, Xianzhi Lan, Tiantian Chen, Qijie Mo, Chao Peng, Jianbo Jia, Jiewei Liu, Cheng‐Yong Su

**Affiliations:** ^1^ School of Environmental and Chemical Engineering Jiangmen Key Laboratory of Synthetic Chemistry and Cleaner Production Institute of Carbon Peaking and Carbon Neutralization Wuyi University Jiangmen P.R. China; ^2^ School of Food & Pharmaceutical Engineering Zhaoqing University Zhaoqing China; ^3^ GBRCE For Functional Molecular Engineering LIFM IGCME School of Chemistry Sun Yat‐Sen University Guangzhou China

**Keywords:** charge‐assisted hydrogen‐bonded organic frameworks, dual‐pathway mechanism, H_2_O_2_ photosynthesis, heterogeneous catalysis, multiple charge transfer channels

## Abstract

The production of H_2_O_2_ via artificial photosynthesis is often limited by inefficient charge‐carrier separation, causing significant charge recombination and slow electron transfer. Herein, we report a facile strategy to regulate the photogenerated charge carrier transportation in hydrogen‐bonded organic frameworks (HOFs) via the construction of charge‐assisted hydrogen bonds. Specifically, the amount of charge transfer channels in the pyrene‐based HOFs can be modulated from one in FDU‐HOF‐3 to two in WYU‐HOF‐1 (WYU = Wuyi University) and three in WYU‐HOF‐2, as evidenced by the in situ characterization and theoretical calculations, leading to the highest separation/transfer efficiency in WYU‐HOF‐2. In addition, the fluorine‐functionalized WYU‐HOF‐2 also contributes to the enhanced charge migration and separation. Catalytic studies reveal that WYU‐HOF‐2 shows the highest H_2_O_2_ generation rate from water, oxygen, and light without sacrificial agents, which is 3.4‐ and 62‐fold higher than that of WYU‐HOF‐1 and FDU‐HOF‐3, respectively. Mechanistic studies disclose that both of WYU‐HOF‐1 and WYU‐HOF‐2 facilitate photocatalytic H_2_O_2_ production via the 2e^−^ oxygen reduction reaction (ORR) and the 2e^−^ water oxidation reaction (WOR) pathways. This work offers a promising strategy for regulating charge carrier transport in HOFs, thereby enhancing their photocatalytic performance.

## Graphical abstract

This work reports a strategy of engineering charge‐assisted hydrogen bonds to tailor charge transfer channels in HOFs, significantly improving their performance in photocatalytic H_2_O_2_ production.

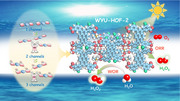

## 1 Introduction

Hydrogen peroxide (H_2_O_2_), valued for its storage convenience and high energy density, serves as a widely utilized eco‐friendly oxidant across diverse sectors such as chemical synthesis, textile manufacturing, and wastewater treatment [1, 2]. This broad applicability drives substantial annual demand. However, conventional H_2_O_2_ production methods, notably the anthraquinone (AQ) oxidation process and direct synthesis employing noble metal catalysts, exhibit significant limitations [3, 4]. These include high energy consumption, generation of substantial wastewater, elevated operational costs, and often unsatisfactory selectivity. Consequently, there is a compelling need to develop alternative production strategies that are energy‐efficient, cost‐effective, and operable under milder conditions. Artificial photosynthesis, utilizing sunlight, water, and oxygen to generate H_2_O_2_ without sacrificial reagents or external energy input, represents a highly promising sustainable method for H_2_O_2_ production [5]. Various photocatalysts, including graphitic carbon nitride (g‐C_3_N_4_) [6], metal–organic frameworks (MOFs) [7], covalent organic frameworks (COFs) [8], and organic polymers [9], have been widely employed for photocatalytic H_2_O_2_ production. Nevertheless, the practical viability of this process is often compromised by detrimental charge dynamics in photocatalysts, namely rapid electron‐hole recombination and insufficient charge transfer, which critically constrict the efficiency of H_2_O_2_ photosynthesis.

As an emerging class of porous crystalline materials, hydrogen‐bonded organic frameworks (HOFs) are assembled through hydrogen bonding between organic monomers, in conjunction with secondary intermolecular interactions like *π*–*π* stacking and van der Waals forces [10, 11, 12]. Owing to their low‐energy synthesis, low toxicity, unique solution processability, as well as molecular‐level designability, HOFs have garnered considerable interest as novel photocatalysts [13, 14, 15]. Moreover, the ordered stacking of extended *π*‐conjugated structural units and the resulting *π*–*π* interactions could serve as electron‐transfer channels along the stacking direction, facilitating efficient charge transfer, thereby boost the photocatalytic activity [16, 17]. This enables the application of HOFs in diverse artificial photocatalytic processes, including CO_2_ reduction [18] and H_2_ evolution [19]. However, HOFs as photocatalysts for H_2_O_2_ generation were rare studied. Very recently, Zhong and co‐workers pioneered the application of hydrogen‐bonded organic frameworks (HOFs) as photocatalysts for H_2_O_2_ photosynthesis [20]. So far, various strategies, such as loading metal nanoparticles [21], constructing heterojunctions [22], and tuning the molecular *π*–*π* packing [23] have been employed to enhance charge transfer and separation in HOFs. However, these approaches often involve complex fabrication procedures, which can make them relatively time‐ and energy‐intensive. Consequently, developing a facile and cost‐effective strategy to improve charge transfer of HOFs for boosting H_2_O_2_ photosynthesis is highly desirable.

Charge‐assisted hydrogen bonds are hydrogen bonds in which the donor is positively charged or the acceptor is negatively charged [21, 24, 25, 26, 27, 28, 29, 30]. The introduction of charge‐assisted hydrogen bonds in HOFs not only provides electrostatic stabilization to the frameworks, thereby enhancing their stability [31, 32], but more significantly, the hydrogen bonds can serve as atomic‐level charge transfer channels that facilitate rapid charge migration [33, 34]. Hence, the fabrication of multiple charge transfer channels via charge‐assisted hydrogen bonds offers a promising strategy to overcome the aforementioned challenge and boost the H_2_O_2_ photosynthesis efficiency of HOFs.

**FIGURE 1:**
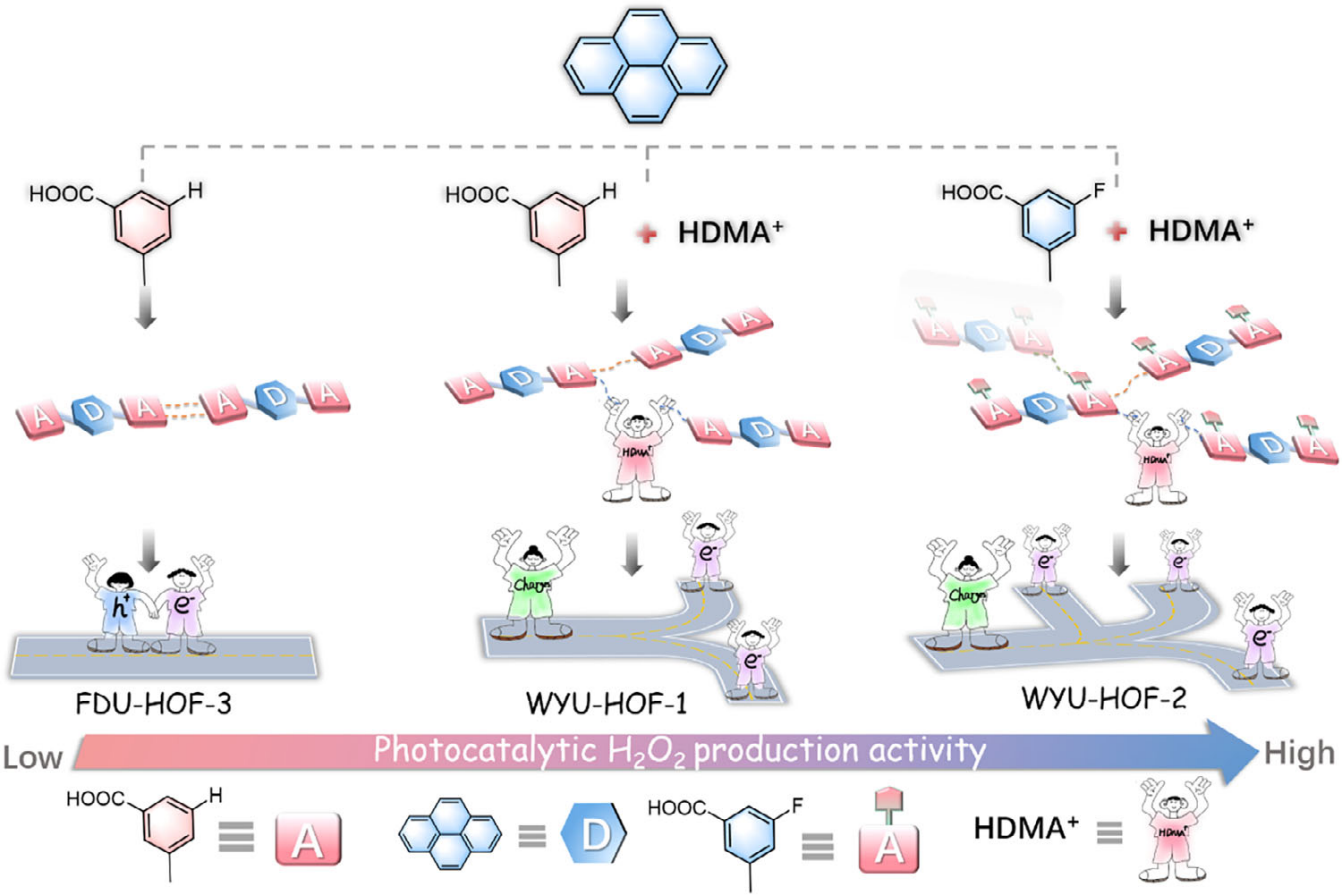
Schematic diagram illustrating the synthesis of charge‐assisted HOFs and the modulation of their charge‐transfer channels to boost H_2_O_2_ production.

Herein, we demonstrate that the construction of charge‐assisted hydrogen bonds provides a straightforward and effective strategy to tailor charge transfer pathways in pyrene‐based HOFs, thereby facilitating the transport of photogenerated charge carriers. A series of pyrene‐based HOFs (namely WYU‐HOF‐1 and WYU‐HOF‐2) with charge‐assisted hydrogen bonds have been successfully synthesized (Figure 1). For comparison, the reported pyrene‐based HOF without charge‐assisted hydrogen bonds (namely FDU‐HOF‐3) is also synthesized as a reference [35]. Combining in situ characterization with theoretical calculations, we find that these three HOFs exhibit electron donor‐acceptor characteristics, with the pyrene moiety functioning as the donor and the benzoate group as the acceptor. Thus, one, two, and three distinct charge transfer channels are identified in FDU‐HOF‐3, WYU‐HOF‐1, and WYU‐HOF‐2, respectively. Moreover, fluorine incorporation into the framework serves to enhance the separation efficiency of photogenerated charge carriers while simultaneously enabling an additional charge transfer pathway. By effectively facilitating the transport of photogenerated charge carriers, this structural control strategy significantly boosts the photocatalytic production of H_2_O_2_ from water and O_2_ under visible light, via a dual‐channel ORR/WOR mechanism.

## 2 Results and Discussion

### 2.1 Synthesis, Structure and Characterization

**FIGURE 2:**
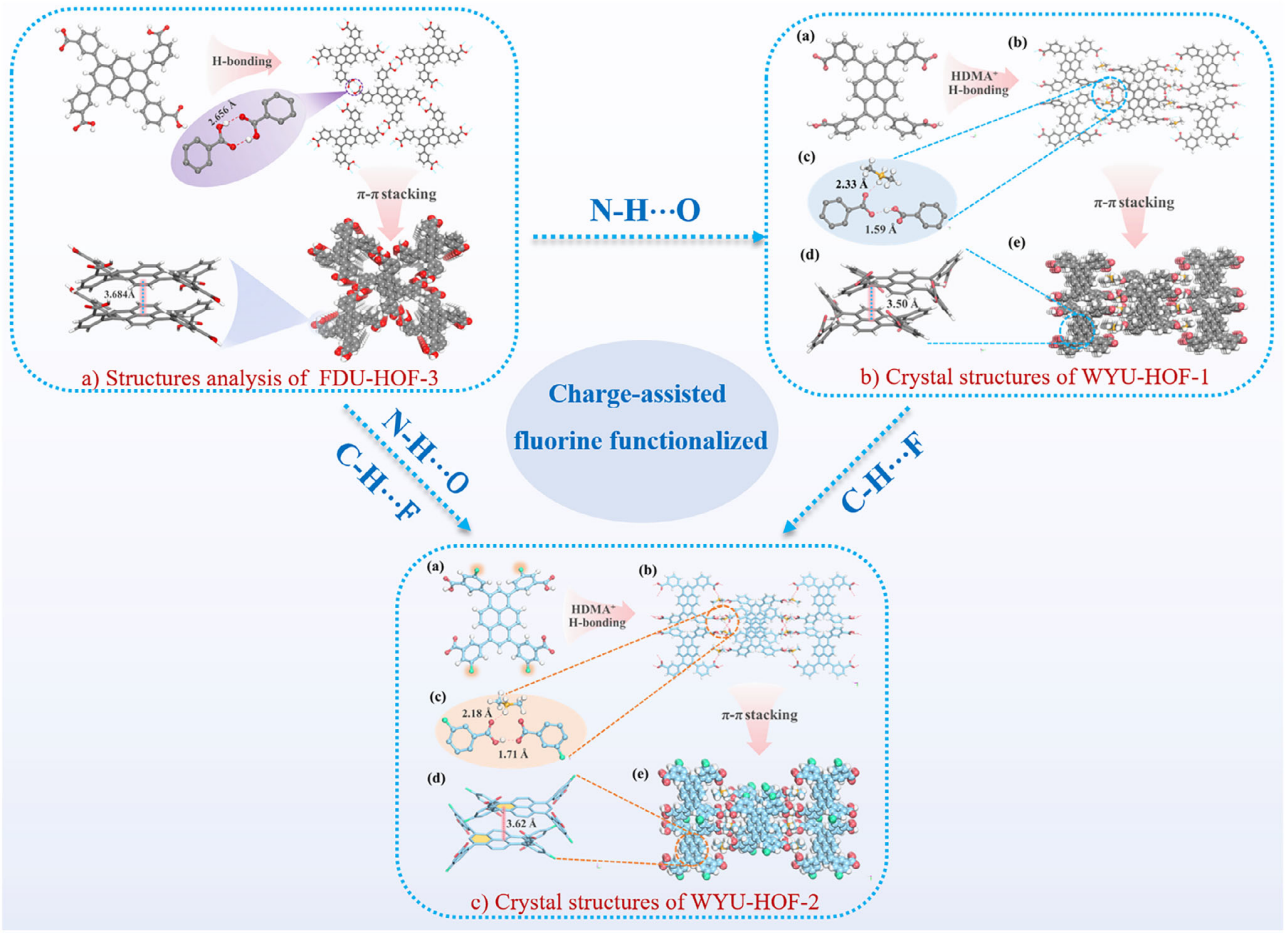
HOFs crystal structure. Schematic structure of (a) FDU‐HOF‐3; (b) WYU‐HOF‐1; (c) WYU‐HOF‐2. (C: gray/blue; O: red; H: White; N: Yellow; F: Green).

The 1,3,6,8‐tetrakis(3‐carboxyphenyl) pyrene and 1,3,6,8‐tetrakis(3‐fluoro‐5‐carboxyphenyl) pyrene, denoted as H_4_PTTB‐R (R = H, F), are synthesized according to the literatures with slight modification (Scheme S1) [35, 36, 37, 38]. Solvothermal reaction of H_4_PTTB‐R in a DMF/H_2_O/HNO_3_ mixture at 100°C for 72 h affords yellow rod‐shaped crystals of WYU‐HOF‐1 and WYU‐HOF‐2, respectively (Figure S1), where the decomposition of DMF generates dimethylamine cation ([NH_2_(CH_3_)_2_]^+^, namely HDMA^+^) as an in situ‐formed base. This deprotonates two carboxylic groups of H_4_PTTB‐R (R = H, F), thus enabling the formation of charge‐assisted HOF. The FT‐IR spectra of WYU‐HOF‐1 and WYU‐HOF‐2 are displayed in Figure S7. To facilitate comparison and elucidate the role of charge‐assisted H‐bonding, the reference HOF, namely FDU‐HOF‐3, which is also assembled from the H_4_PTTB ligand, is employed in this study (Figure 2a) [35].

Single‐crystal X‐ray diffraction (SCXRD) analyses reveal that WYU‐HOF‐1 crystallizes in the monoclinic system *I*2/*a* space group, while WYU‐HOF‐2 adopts the *C*2/*c* space group (Tables S1 and S2). The asymmetric unit of both structures comprises half a molecule of H_2_PTTB^2−^ anion (or fluorinated H_2_PTTB^2−^‐F) and one dimethylamine cation (HDMA^+^) (Figures S2 and S3). In WYU‐HOF‐1, each H_2_PTTB^2−^ unit connects with four neighboring H_2_PTTB^2−^ and four HDMA^+^ (Figure S2). Adjacent H_2_PTTB^2−^ are connected through strong intermolecular O─H⋯O hydrogen bonds (with O⋯H distance of 1.59 Å, O⋯O distance of 2.50 Å and O─H⋯O bond angle of 173.7°), generating a 2D layered rhombic network, meanwhile one HDMA^+^ connects with one deprotonated carboxyl group and one carboxyl group coming from different H_2_PTTB^2−^ units through charge‐assisted N─H⋯O hydrogen bonds (with H⋯O distances of 2.36 Å) (Figure 2b). Topologically, each H_2_PTTB^2−^ unit acts as an eight‐connected (8‐c) node, while each HDMA^+^ unit serves as a two‐connected (2‐c) node, which establishes a 2,8‐connected framework with point symbol of {4 [6]·6 [18]·10^4^}{4} [2] (Figure S5). In FDU‐HOF‐3, the H_4_PTTB units are connected in a head‐to‐head orientation via intermolecular hydrogen bonding within the same plane, along with strong *π*–*π* stacking interactions between adjacent planes, which facilitates the formation of 4,4‐connected framework with ***sql*** topology (Figure 2a; Figure S4). The introduction of assisted hydrogen bonding in WYU‐HOF‐1 induces a distinct spatial conformation compared with that in FDU‐HOF‐3 (Figure 2a,b) [35].

WYU‐HOF‐2 features an iso‐reticular, charge‐assisted framework assembled via similar yet more distorted O─H⋯O bonds (with O⋯H distance of 1.71 Å, O⋯O distance of 2.50 Å, O─H⋯O bond angle of 157.5°), and exhibits the same topology as WYU‐HOF‐1 (Figure 2c; Figure S6 and Table S3). It is noted that C–H···F interactions are formed in the WYU‐HOF‐2 with H⋯F distances ranging from 2.7 to 3.2 Å (Figure S3). In both the structures of WYU‐HOF‐1 and WYU‐HOF‐2, multiple *π*–*π* stacking interactions exist between the adjacent pyrene rings, and the distances of centroid‐centroid are shown in Figure 2b,c. By virtue of these hydrogen‐binding synthons (Figures S2 and S3), the layered rhombic networks adopt a stacking mode and further extended into 3D supramolecular frameworks through *π*–*π* stacking interactions and N─H⋯O hydrogen bonds between HDMA^+^ cations and H_2_PTTB^2^^−^ anions (Figure 2b,c).

The good agreement between the experimental and simulated PXRD patterns indicates the purity of the as‐synthesized WYU‐HOF‐1 and WYU‐HOF‐2 (Figures S8 and S9). Scanning electron microscopy (SEM) reveals the rectangular prism morphology of WYU‐HOF‐1 and WYU‐HOF‐2 (Figures S10 and S11), whereas energy‐dispersive X‐ray spectroscopy (EDS) mappings and full X‐ray photoelectron spectroscopy (XPS) spectra confirm their elemental compositions (Figures S12–S14). The observed differences in composition between WYU‐HOF‐1 and WYU‐HOF‐2 align with their distinct crystal structures. Thermogravimetric analysis (TGA) curves reveal the high thermal stability for both WYU‐HOF‐1 and WYU‐HOF‐2 (Figure S15), exhibiting negligible mass loss up to 300°C and 250°C, respectively. Both WYU‐HOF‐1 and WYU‐HOF‐2 demonstrate high chemical stability. Their crystalline structures maintain integrity after 7 days of soaking in various organic solvents and aqueous solutions with different pH values (1‐14 for WYU‐HOF‐1 and 1–11 for WYU‐HOF‐2), with no significant changes in peak profiles observed (Figures S16–S19). The excellent stability of WYU‐HOF‐1 and WYU‐HOF‐2 stems from the combined effects of charge‐assisted H‐bonding, *π*–*π* interactions, and other weak forces.

### 2.2 Boosting Charge Transfer in HOFs by Harnessing Multiple Transport Channels

**FIGURE 3:**
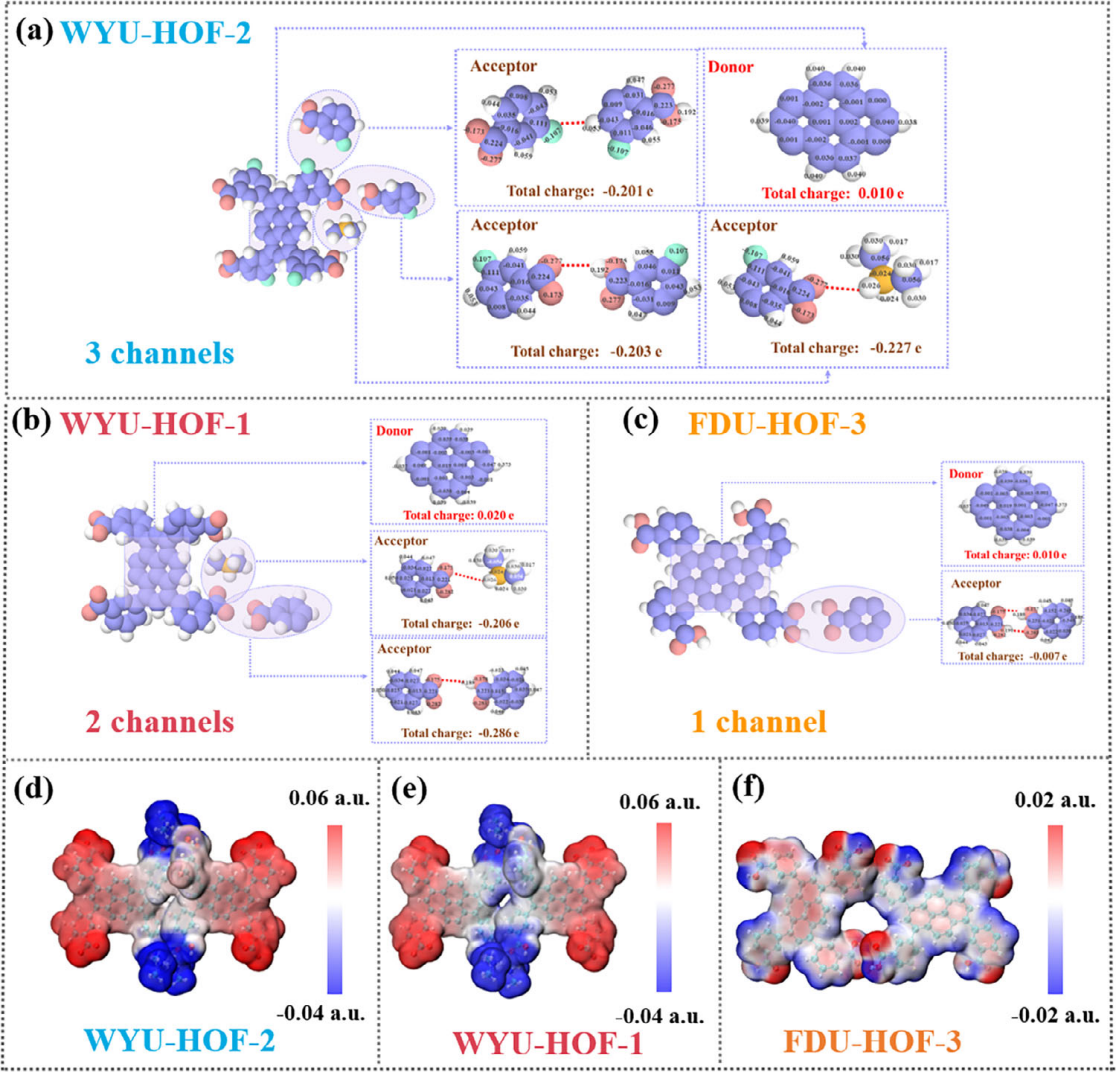
Hirshfeld charge distribution in WYU‐HOF‐2 (a), WYU‐HOF‐1 (b) and FDU‐HOF‐3 (c); Electrostatic potential surface of WYU‐HOF‐2 (d), WYU‐HOF‐1 (e) and FDU‐HOF‐3 (f).

The Hirshfeld charge distribution analysis based on DFT calculations indicates that the electron‐rich pyrene moiety in all three HOFs can serve as electron donor units, while the combination of two benzoate groups from two adjacent ligands, along with the pairing of a benzoate group and an HDMA^+^ cation, both exhibit electron‐withdrawing characteristics and can serve as electron acceptors (Figure 3a–c). In addition, the introduction of fluorine in the H_4_PTTB‐F moiety creates another electron acceptor, formed by the pairing of fluorinated benzoate groups from two adjacent H_4_PTTB‐F ligands. Notably, the hydrogen bonds existed between these two components can serve as atomic‐level charge transfer channels, thereby facilitating rapid charge migration [33]. Accordingly, FDU‐HOF‐3, WYU‐HOF‐1, and WYU‐HOF‐2 possess one, two, and three distinct types of donor‐acceptor combinations, respectively (Figure 3a–c). In other words, there are one, two and three channels for charge transfer in FDU‐HOF‐3, WYU‐HOF‐1 and WYU‐HOF‐2, respectively (Figure 1). Therefore, through the deliberate design of charge‐assisted hydrogen bonds in the HOF structures, the pathways for charge carrier transport can be fine‐tuned. This optimization is expected to markedly enhance charge carrier dynamics and promote the overall photocatalytic reaction kinetics.

Further electrostatic potential (ESP) analysis reveals that the H_4_PTTB‐H and H_4_PTTB‐F moieties serve as the primary negative potential regions in WYU‐HOF‐1 and WYU‐HOF‐2, respectively (Figure S20), and thus constitute the principal sites for photoexcited carrier generation. Furthermore, the strong electron‐withdrawing character of the fluorine atom in H_4_PTTB‐F induces an additional negative ESP center, resulting in more negative potentials throughout the entire WYU‐HOF‐2 framework compared to WYU‐HOF‐1 and FDU‐HOF‐3 (Figure 3d–f).

**FIGURE 4:**
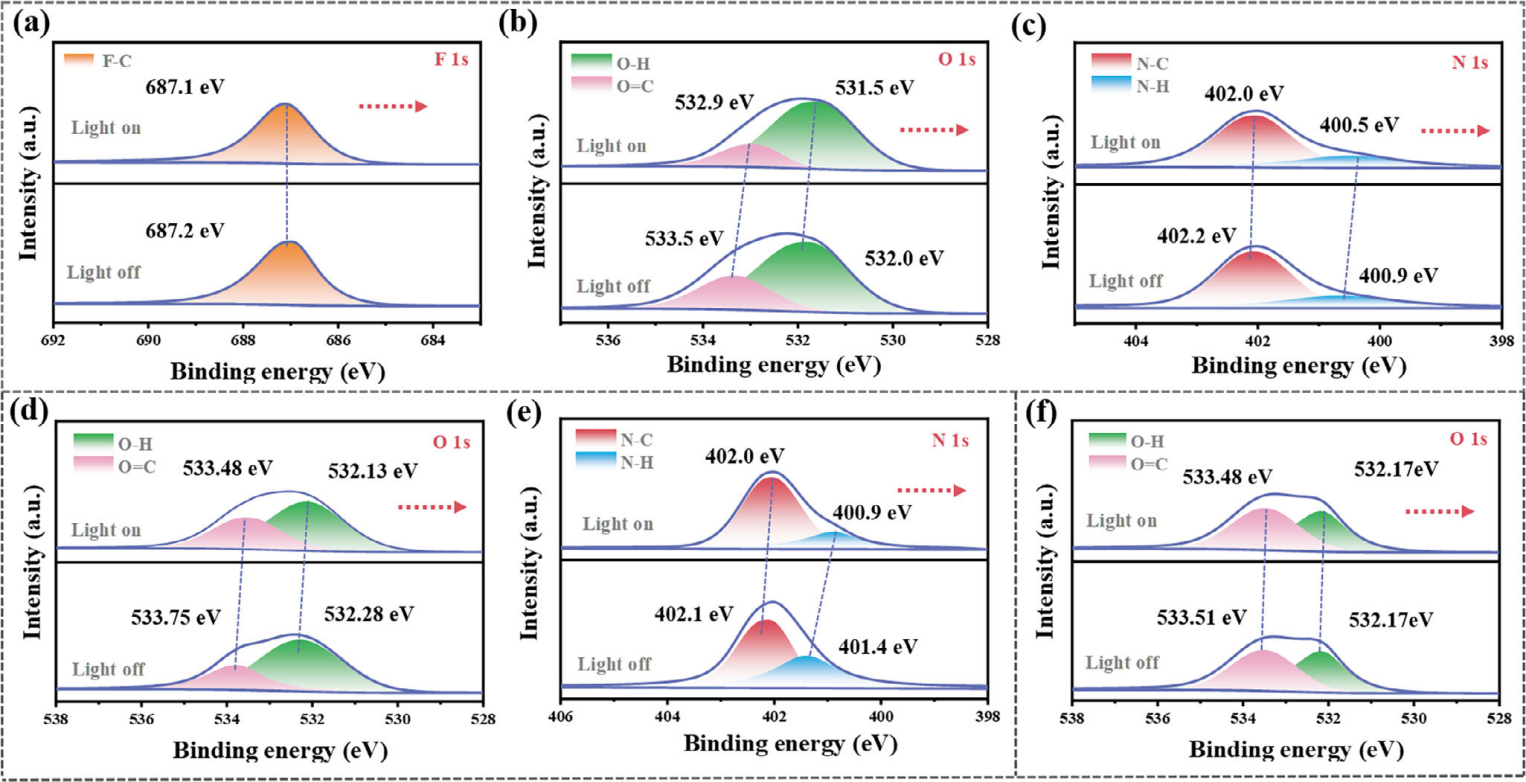
ISI‐XPS for F 1s (a), O 1s (b) and N 1s (c) of WYU‐HOF‐2; O 1s (d) and N 1s (e) of WYU‐HOF‐1; O 1s (f) of FDU‐HOF‐3.

To validate the multiple charge transfer channels in the HOFs, in situ irradiated X‐ray photoelectron spectroscopy (ISI‐XPS) experiments were carried out [39]. Under visible light irradiation, the F 1s, O 1s, and N 1s spectra of WYU‐HOF‐2 all shift toward lower binding energies (Figure 4a–c). A similar trend was observed for WYU‐HOF‐1, where the O 1s and N 1s peaks also shift to lower binding energies upon illumination (Figure 4d,e). Likewise, the O 1s spectrum of FDU‐HOF‐3 exhibits a corresponding shift toward lower binding energy following visible light exposure (Figure 4f). These observations indicate that the F, O, and N atoms synergistically function as electron accumulation sites, thereby directly validating the proposed charge transfer channels, which is in line with the above Hirshfeld charge distribution analysis.

**FIGURE 5:**
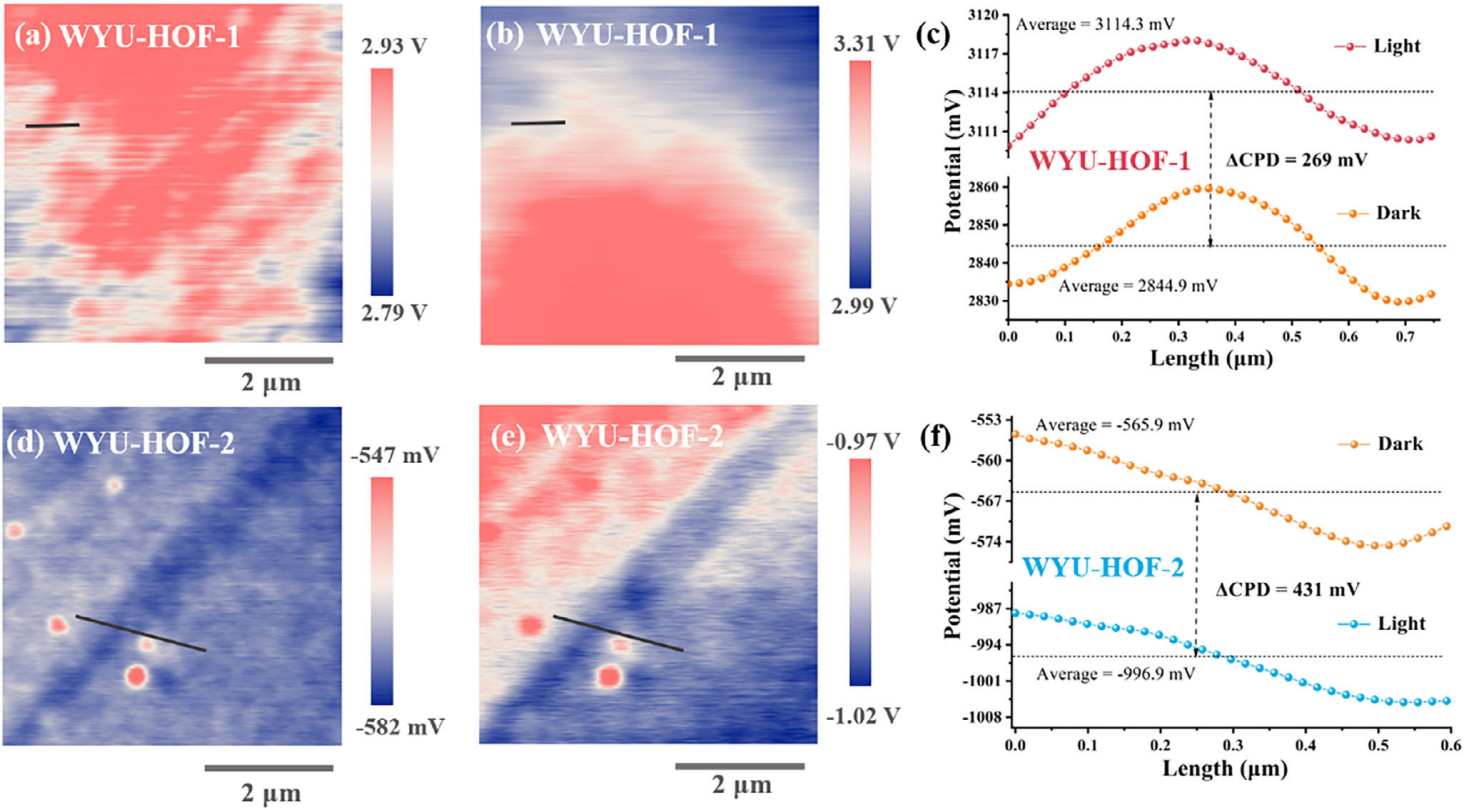
KPFM images of WYU‐HOF‐1 in the dark (a) and under illumination (b); (c) corresponding line‐scanning surface potential profile of WYU‐HOF‐1 in the absence and presence of light; KPFM images of WYU‐HOF‐2 in the dark (d) and under illumination (e); (f) corresponding line‐scanning surface potential profile of WYU‐HOF‐2 in the absence and presence of light.

To directly visualize the charge migration processes, light‐assisted Kelvin probe force microscopy (KPFM) was used to characterize the surfaces of WYU‐HOF‐1 and WYU‐HOF‐2. Upon formation of an electrical contact, charge redistributes between the sample and the Kelvin tip until their Fermi energy levels ((*E*_f_) equalize, thereby reaching electronic equilibrium [40]. Consequently, the contact potential difference (CPD) arises directly from the difference between the work functions of the tip (*Φ*_t_) and the sample (*Φ*_s_). As shown in Figure 5a,b,d,e, the color contrast arises from the material‐dependent CPD values, which are caused by light‐induced shifts in the *E*_f_. Upon illumination, the generation and separation of photoexcited electron‐hole pairs induces a downward bending of the *E*_f_ at the surface. This is equivalently described as an upward bending of the vacuum level (*E*_v_), which consequently caused a decrease in the CPD values. Figure 5c,f shows the CPD variations measured along a straight line for WYU‐HOF‐1 and WYU‐HOF‐2, respectively, comparing the results before and after visible light illumination. As reported in the literature [41], the observed contact potential difference (ΔCPD) in the absence and presence of light serves as a direct metric for quantifying the surface photovoltage effect. Therefore, the larger ΔCPD observed in WYU‐HOF‐2 (431 mV) compared to WYU‐HOF‐1 (269 mV) indicates more efficient charge separation and migration. This enhancement is primarily attributed to the modified dielectric environment and the introduction of shallow trap states resulting from fluorination, which prolong carrier lifetime.

**FIGURE 6:**
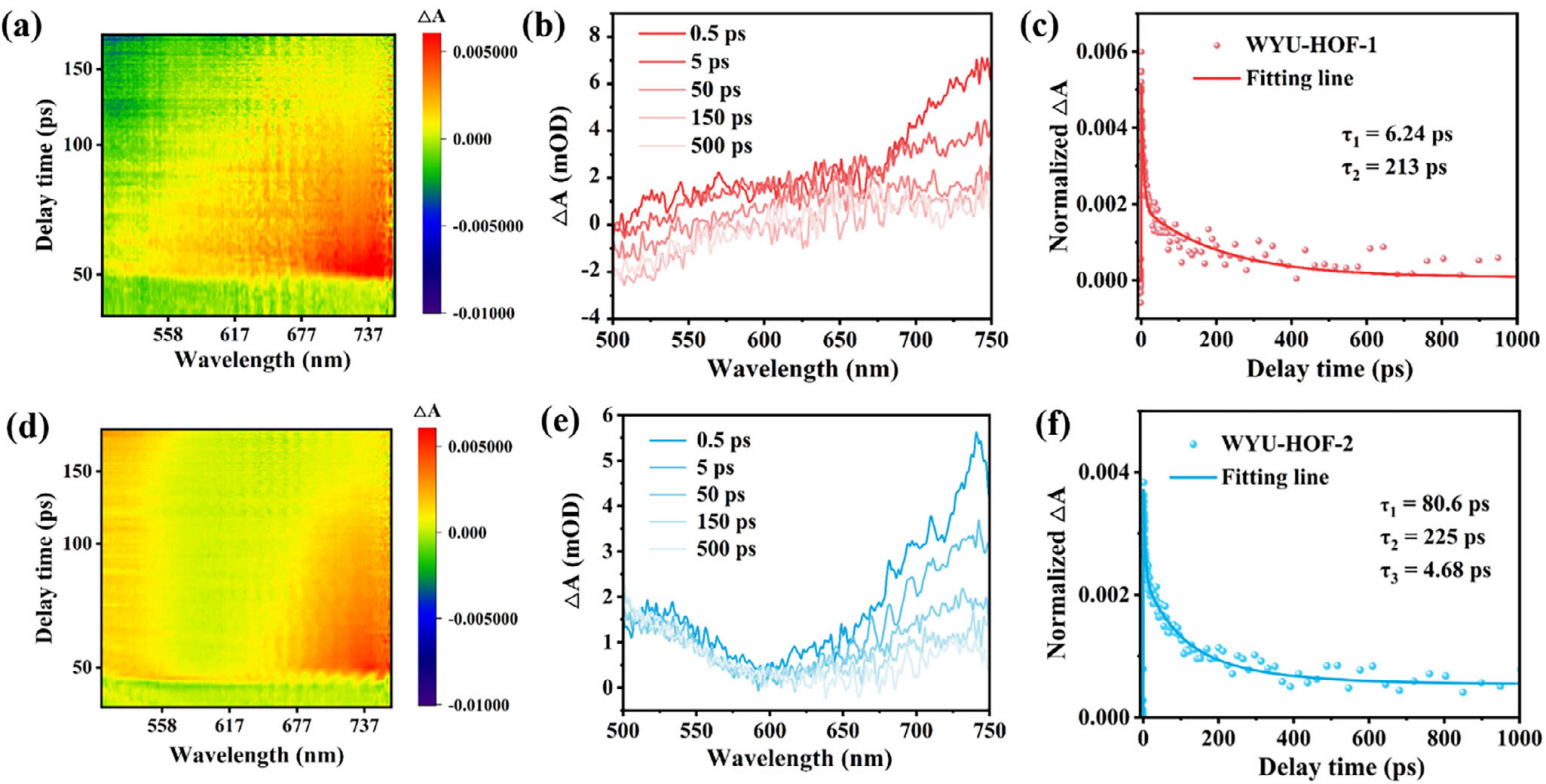
2D transient absorption surface plots of (a) WYU‐HOF‐1 and (d) WYU‐HOF‐2. The transient absorption at different decay times of (b) WYU‐HOF‐1 and (e) WYU‐HOF‐2, and corresponding decay kinetics curves of (c) WYU‐HOF‐1 (at 720 nm) and (f) WYU‐HOF‐2 (at 720 nm).

Moreover, femtosecond time‐resolved transient absorption spectroscopy (fs‐TAS) was employed to investigate the photoexcited carrier dynamics of WYU‐HOF‐1 and WYU‐HOF‐2 (Figure 6). Femtosecond transient absorption spectroscopy (fs‐TAS) with a 420 nm pump pulse reveals that both HOFs exhibit positive photoinduced absorption (PIA) bands in the visible region. Time‐resolved transient absorption spectra further show that the PIA signal decays more slowly in WYU‐HOF‐2, indicating longer carrier lifetimes and more efficient charge separation (Figure 6b,e). The decay kinetics of WYU‐HOF‐1 and WYU‐HOF‐2 were fitted at 720 nm using biexponential and triexponential functions, respectively. As shown in Figure 6c,f, the τ_1_ values for WYU‐HOF‐1 and WYU‐HOF‐2 are 6.24 and 80.6 ps, respectively, while the corresponding τ_2_ values are 213 and 225 ps. Compared with WYU‐HOF‐1, both τ_1_ and τ_2_ are substantially extended in WYU‐HOF‐2, indicating markedly slower carrier decay. These results provide direct evidence that the multiple charge transfer channels in WYU‐HOF‐2 effectively suppress charge recombination and prolong the lifetime of photogenerated carriers. This extended lifetime allows more sufficient reaction time for surface catalytic processes, which is consistent with the superior photocatalytic performance observed for WYU‐HOF‐2.

### 2.3 Photoelectric Property and Photocatalytic H_2_O_2_ Performance

**FIGURE 7:**
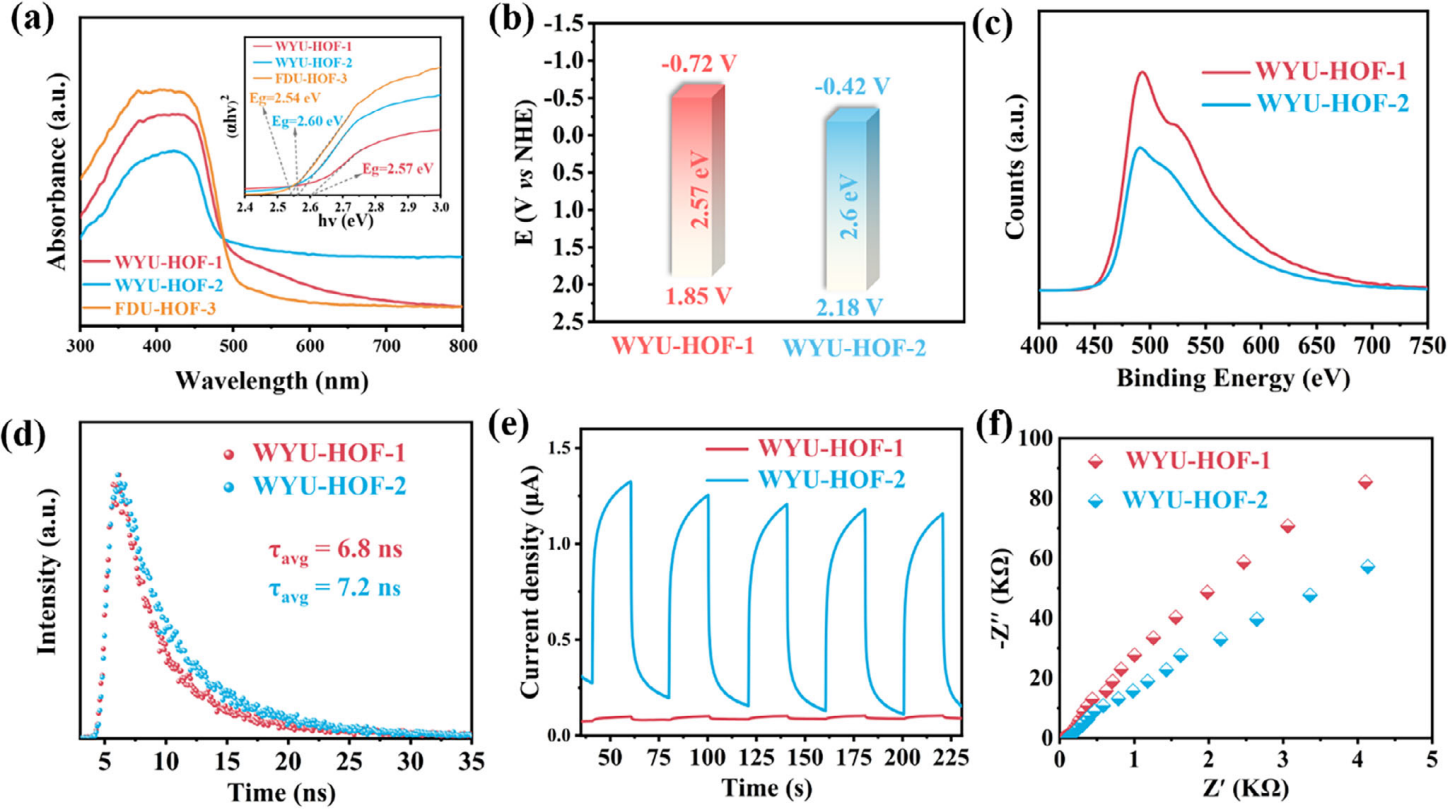
Spectroscopy and electrochemical characterisation of WYU‐HOF‐1 and WYU‐HOF‐2. (a) solid‐state UV–vis spectra and Tauc plots (inset) of the three HOFs; (b) energy band structure; (c) the steady‐state photoluminescence (PL) measurements; (d) time‐resolved photoluminescence spectra; (e) transient photocurrent response; (f) EIS Nyquist diagram.

The light‐harvesting properties of as‐synthesized HOFs are characterized by the solid‐state UV–vis spectra. As shown in Figure 7a, a broad light absorption ranging from 300 to 700 nm is observed, which indicated the well visible light absorption performance of the three HOFs. The bandgaps of WYU‐HOF‐1, WYU‐HOF‐2 and FDU‐HOF‐3 are calculated to be 2.57, 2.6 and 2.54 eV, respectively, as determined by the Tauc plots (Figure 7a, insert). Mott‐Schottky (M−S) measurements are further performed to determine the energy levels of the materials. As depicted in Figure S21, the M−S plots of WYU‐HOF‐1 display a positive slope, indicating the *n*‐type semiconductor characteristics of WYU‐HOF‐1; whereas WYU‐HOF‐2 shows a negative slope, characteristic of a p‐type semiconductor (Figure S22) [38]. The conduction band (CB) values are −1.3 and 1.6 V vs Ag/ AgCl at pH 6.5 of WYU‐HOF‐1 and WYU‐HOF‐2. Relative to the normal hydrogen electrode (NHE) at pH 0 are −0.72 and −0.42 V, respectively (via Equations S1 and S2). Then, the valence band (VB) values of WYU‐HOF‐1 and WYU‐HOF‐2 are calculated to be 1.85 and 2.18 V, respectively. Based on the above results, the band structures for WYU‐HOF‐1 and WYU‐HOF‐2 can be derived (Figure 7b). These results demonstrate the thermodynamic feasibility for both HOFs to simultaneously drive H_2_O_2_ photosynthesis via O_2_ reduction (E (O_2_/H_2_O_2_) = −0.33 V vs NHE, pH 0) and H_2_O oxidation (E (H_2_O/H_2_O_2_) = +1.76 V vs NHE, pH 0) [42]. Therefore, the favorable energy band structures of these HOFs indicate that they are thermodynamically feasible for efficient H_2_O_2_ photosynthesis via the coupled ORR and WOR pathways.

**FIGURE 8:**
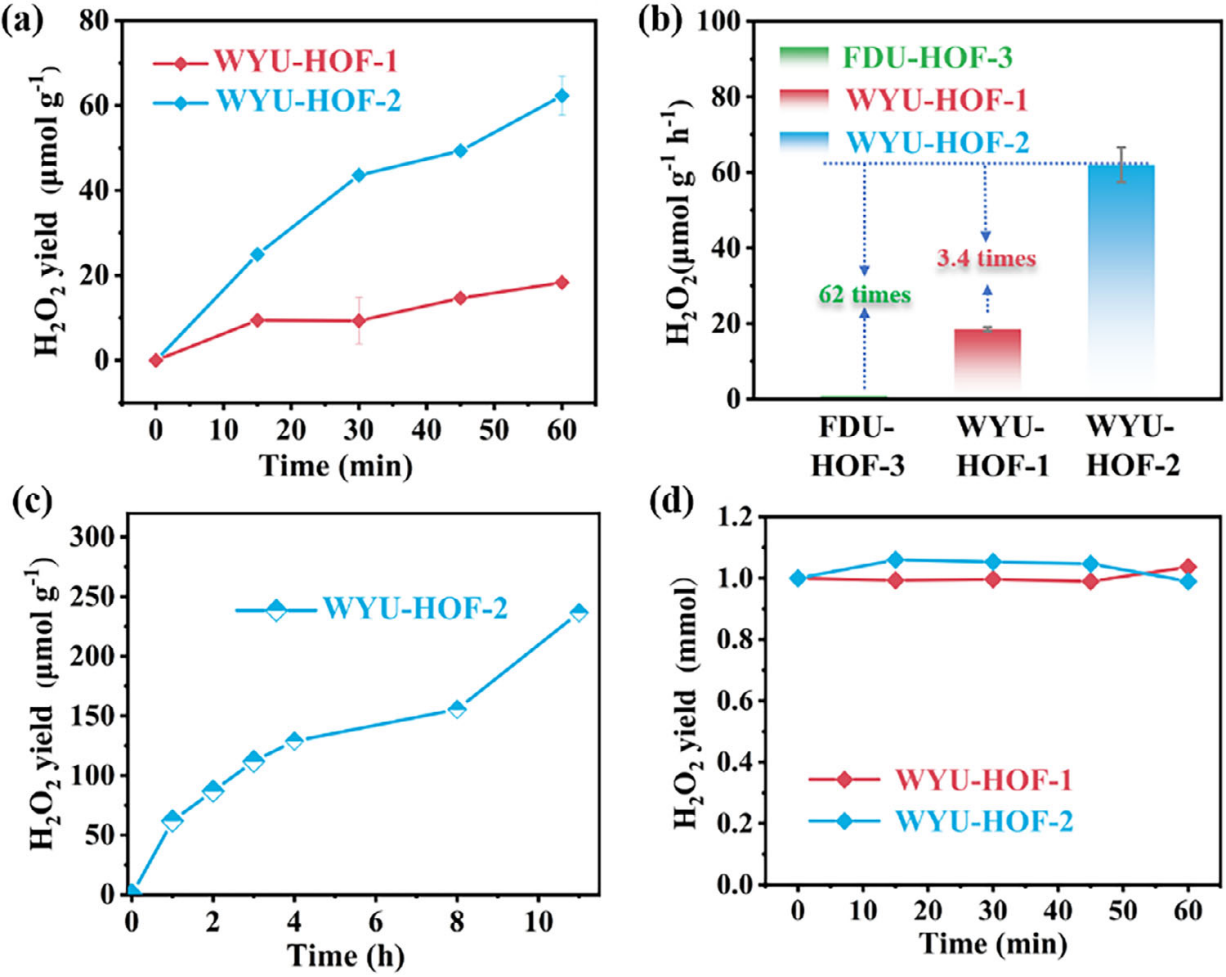
(a) Time‐dependent H_2_O_2_ photogeneration by WYU‐HOF‐1 and WYU‐HOF‐2, respectively (λ > 420 nm; 300 W xenon lamp; 10 mg catalyst in 10 mL pure water; O_2_‐saturated, 25°C); (b) Photocatalytic H_2_O_2_ production by various photocatalysts: FDU‐HOF‐3, WYU‐HOF‐1 and WYU‐HOF‐2; (c) the photogenerated H_2_O_2_ concentration of WYU‐HOF‐2 with prolonged irradiation time over 11 h; (d) H_2_O_2_ (1 mM) degradation study by WYU‐HOF‐1 and WYU‐HOF‐2 upon illumination.

Subsequently, the photogenerated charge separation and migration capabilities of WYU‐HOF‐1 and WYU‐HOF‐2 are investigated using photoluminescence (PL) spectroscopy, time‐resolved photoluminescence (TRPL), transient photocurrent measurements, and electrochemical impedance spectroscopy (EIS). The PL spectra reveal the substantially quenched emission in WYU‐HOF‐2 compared to WYU‐HOF‐1 (Figure 7c), indicating effective suppression of photogenerated charge carrier recombination. The TRPL spectra reveals that the photogenerated charge lifetime in WYU‐HOF‐2 is longer than that in WYU‐HOF‐1 (Figure 7d). This extended lifetime underscores the critical role of fluorine functionalization in facilitating charge migration and separation, which effectively suppresses charge recombination and leads to the formation of long‐lived charge carriers. Photocurrent and EIS tests further corroborate these findings. WYU‐HOF‐2 exhibits a higher photocurrent density than WYU‐HOF‐1, implying the higher charge‐transfer efficiency (Figure 7e). Moreover, WYU‐HOF‐2 demonstrates a lower charge‐transfer resistance, as evidenced by the smaller semicircle in its EIS Nyquist plot (Figure 7f). The collective evidence from PL, TRPL, photocurrent, and EIS measurements consistently demonstrate that increasing the number of charge transfer pathways in HOFs effectively facilitates the separation of photogenerated charge carriers and enhances charge‐carrier mobility,

The photocatalytic H_2_O_2_ generation experiments are conducted in pure water under an O_2_ atmosphere without adding any co‐catalysts, photosensitizers, or sacrificial agents. The production of H_2_O_2_ is quantified using the DPD method [43]. As shown in Figure 8a, the H_2_O_2_ concentration gradually increases with prolonged illumination time. WYU‐HOF‐1 exhibits an H_2_O_2_ production rate of 18.5 µmol g^−^^1^ h^−^^1^. Upon incorporation of fluorine groups, the photocatalytic H_2_O_2_ production rate of the resulting WYU‐HOF‐2 increases markedly to 62 µmol g^−^^1^ h^−^^1^, which is more than three times that of WYU‐HOF‐1 (Figure 8b). This trend correlates with the efficiency of photogenerated charge carrier separation in both HOFs. Notably, only a trace amount of H_2_O_2_ is detected in the FDU‐HOF‐3 photocatalytic system. These results demonstrate that enhancing the number of charge transfer pathways and the fluorination‐induced modification of framework can effectively improve the production efficiency of H_2_O_2_. The apparent quantum yield (AQY) of WYU‐HOF‐2 is calculated to be 0.06% at 420 nm (Figure S24). The long‐term irradiation experiment reveals that the H_2_O_2_ production yield of WYU‐HOF‐2 increased continuously throughout the entire 11 h period, reaching a final yield of 250 µmol g^−^^1^ (Figure 8c). Degradation tests under N_2_ atmosphere reveals that negligible H_2_O_2_ decomposition occurred for both WYU‐HOF‐1 and WYU‐HOF‐2 over the photocatalytic process (Figure 8d). The effects of different water sources (river water, lake water, and tap water) on the H_2_O_2_ production performance of WYU‐HOF‐2 are also investigated, and the results show a decreased yield (Figure S25), attributed to the inhibitory effects of coexisting organic matter and inorganic ions. Additionally, recycle experiments show that WYU‐HOF‐2 can be reused for 5 runs without significant loss of activity (Figure S26), meanwhile the structural and morphological integrity of WYU‐HOF‐1 and WYU‐HOF‐2 after photocatalysis are well maintained, as indicated by PXRD and SEM analysis (Figures S27–S30).

### 2.4 Photocatalytic H_2_O_2_ Mechanism

**FIGURE 9:**
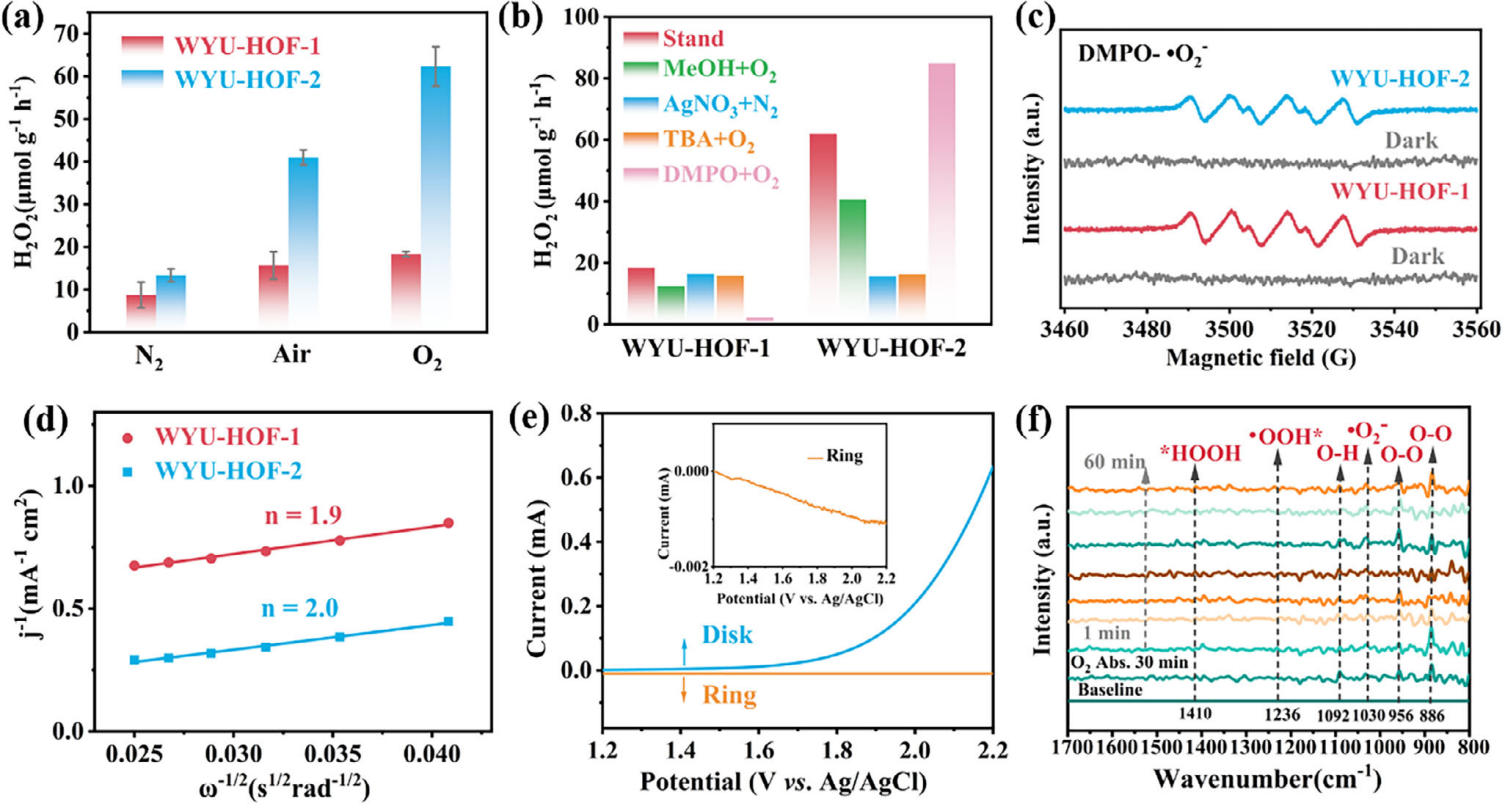
(a) Photocatalytic H_2_O_2_ production activity under various conditions (N_2_, Air, and O_2_); (b) amount of H_2_O_2_ generated in the presence of different scavengers under irradiation for 1 h (MeOH 4% V/V; AgNO_3_ 2 mm; TBA 4% V/V; DMPO 4% V/V); (c) EPR spectra of DMPO‐•O_2_^−^ for WYU‐HOF‐1 and WYU‐HOF‐2; (d) the Koutecký–Levich (K–L) plots obtained via RDE measurements in phosphate buffer (pH 7) solution with continuous O_2_ purging. (e) RRDE voltammograms of WYU‐HOF‐2 obtained in phosphate buffer (pH 7) at rotation speed of 1600 rpm. The potential of Pt ring electrode was set at +0.6 V vs Ag/AgCl to detect H_2_O_2_. (f) DRIFTS spectra of WYU‐HOF‐2 under the saturated O_2_ condition in pure water.

To investigate the potential mechanisms of WYU‐HOF‐1 and WYU‐HOF‐2 for the photocatalytic generation of H_2_O_2_, a series of controlled experiments and in situ characterization are carried out. As shown in Figure S31, no H_2_O_2_ generation is observed in the absence of WYU‐HOF‐2 or light, indicating that both the photocatalyst and light are essential for the photocatalytic reaction. Under light irradiation, replacing oxygen with air decreases the H_2_O_2_ yield, indicating the contribution of the ORR to H_2_O_2_ generation (Figure 9a). Furthermore, even when the O_2_ atmosphere is replaced by N_2_, photocatalytic H_2_O_2_ production remains detectable, although at a significantly reduced yield compared to the O_2_ atmosphere. This indicates the involvement of WOR in H_2_O_2_ formation (Figure 9a).

To elucidate the reaction pathways of the ORR and WOR during the photocatalytic H_2_O_2_ production over WYU‐HOF‐1 and WYU‐HOF‐2, quenching experiments targeting active species are performed. The addition of a hole scavenger (MeOH) or an electron scavenger (AgNO_3_) to the reaction system results in a suppression of H_2_O_2_ generation in both WYU‐HOF‐1 and WYU‐HOF‐2 systems (Figure 9b). This observation suggests the concomitant existence of ORR and WOR pathways in both photocatalytic systems.

The 2e^−^ ORR pathway typically proceeds via either a direct single‐step 2e^−^ process or an indirect stepwise 1e^−^ process. In the WYU‐HOF‐1 system, the addition of a superoxide radical scavenger (5,5‐dimethyl‐1‐pyrroline N‐oxide, DMPO) significantly suppresses H_2_O_2_ production, confirming the critical role of the superoxide radical (•O_2_^−^) in H_2_O_2_ formation (Figure 9b). This demonstrates that the H_2_O_2_ generation in WYU‐HOF‐1 predominantly follows the indirect two‐step 1e^−^ ORR mechanism (O_2_ → •O_2_^−^ → H_2_O_2_). Strikingly, in the WYU‐HOF‐2 system, the addition of DMPO led to a significant enhancement in H_2_O_2_ yield. This observation suggests that scavenging •O_2_^−^ redirected the reaction pathway, shifting from the indirect two‐step 1e^−^ ORR mechanism toward the direct single‐step 2e^−^ ORR mechanism (O_2_ + 2e^−^ + 2H^+^ → H_2_O_2_), thereby establishing the dominance of the direct 2e^−^ ORR pathway [40]. Collectively, these results confirm that H_2_O_2_ production in the WYU‐HOF‐2 system involves both the indirect two‐step 1e^−^ ORR pathway and the direct single‐step 2e^−^ ORR pathway, with a shift toward the latter upon •O_2_^−^ removal. A detailed explanation is presented in Figure S32.

To clarify the reaction pathway of WOR half‐reactions, quenching experiments were conducted using *tert*‐butanol (TBA) as a hydroxyl radical (•OH) scavenger. The results revealed that the photocatalytic H_2_O_2_ yield decreased in both the WYU‐HOF‐1 and WYU‐HOF‐2 systems in the presence of TBA compared to the control without scavenger (Figure 9b). This reduction confirms the pivotal role of hydroxyl radicals in the photocatalytic process, indicating that the WOR in both systems proceeds predominantly via an indirect stepwise single‐electron transfer mechanism (H_2_O→ •OH → H_2_O_2_).

Furthermore, the reactive intermediates were characterized using electron paramagnetic resonance (EPR) spectroscopy. Upon adding the spin trap 5,5‐dimethyl‐1‐pyrroline N‐oxide (DMPO) to the WYU‐HOF‐1 and WYU‐HOF‐2 reaction systems and irradiating for 5 min with visible light, distinct characteristic signals corresponding to the DMPO‐•O_2_^−^ and DMPO‐•OH adducts were observed in the spectrum (Figure 9c; Figure S33) [44]. In contrast, no such signals were detected under dark conditions. These results provide direct evidence for the generation of both reactive intermediates (•O_2_^−^ and •OH) during photocatalysis.

Subsequently, the average electron transfer number (n) for H_2_O_2_ production in both HOFs was determined via rotating disk electrode (RDE) experiments [45]. Linear sweep voltammetry (LSV) measurements were performed on HOF‐modified electrodes at various rotation rates (Figures S34 and S35). The corresponding Koutecký–Levich (K–L) plots, analyzed at 0.6 V vs. RHE, revealed the average electron transfer numbers (n) of 1.9 for WYU‐HOF‐1 and 2.0 for WYU‐HOF‐2 during the photocatalytic H_2_O_2_ generation process (Figure 9d). These values indicate that the ORR over both HOFs predominantly proceeds via a desirable 2e^−^ transfer pathway. Furthermore, the *n*‐value of WYU‐HOF‐2 (2.0) is closer to the ideal value of 2 compared to that of WYU‐HOF‐1 (1.9). This enhancement implies that the multichannel charge transfer pathways as well as the introduction of fluorine in WYU‐HOF‐2 significantly improves selectivity in the 2e^−^ ORR pathway, thereby boosting H_2_O_2_ production efficiency [46, 47]

To elucidate the mechanism of the 2e^−^ WOR, rotating ring‐disk electrode (RRDE) measurements were performed under a N_2_ atmosphere (Figure 9e; Figures S36–S38). The disk electrode potential was scanned from 1.2 to 2.2 V (vs Ag/AgCl) at a rate of 10 mV s^−^^1^, while the Pt ring electrode was held at a constant potential of 0.23 V (vs Ag/AgCl) [48]. This ring potential is typically insufficient for the detection of highly reactive 1e^−^ product [49]. As shown in Figure S36, a significant increase in disk current was observed for WYU‐HOF‐2 at potentials exceeding 1.7 V (vs Ag/AgCl, orange line), indicating that the WOR primarily occurs at the disk electrode. Crucially, no corresponding reduction current was detected on the Pt ring electrode, confirming the absence of O_2_ generation via a 4e^−^ WOR pathway (Figure S36). However, when the ring potential was adjusted to +0.6 V (vs Ag/AgCl), a potential suitable for H_2_O_2_ oxidation on Pt ring, a pronounced oxidation current was observed on the ring for WYU‐HOF‐2, attributable to the oxidation of the H_2_O_2_ generated at the disk (Figure 9e). A similar phenomenon was observed for WYU‐HOF‐1 (Figure S37 and S38). Accordingly, the RRDE results, combined with the above RDE findings, demonstrate that both WYU‐HOF‐1 and WYU‐HOF‐2 facilitate photocatalytic H_2_O_2_ production through a dual pathway 2e^−^ mechanism involving both the 2e^−^ ORR and 2e^−^ WOR pathway.

In situ diffuse reflectance infrared Fourier‐transform spectroscopy (DRIFTS) was also employed to identify real‐time reaction intermediates during H_2_O_2_ production (Figure 9f; Figure S39) [50]. Under dark conditions with saturated oxygen and water vapor for the initial 30 min, both spectra of WYU‐HOF‐1 and WYU‐HOF‐2 exhibited only weak vibrational peaks, demonstrating that the reaction did not proceed in the absence of light (Figure 9f; Figure S39). It is noteworthy that with prolonged irradiation time, WYU‐HOF‐1 and WYU‐HOF‐2 exhibits ^*^OOH vibrational band at 1246 and 1236 cm^−^^1^, which corresponds to the formation of an internal peroxide intermediate in the indirect two‐step 1e^−^ ORR pathway (Figure 9f; Figure S39) [51]. In addition, WYU‐HOF‐2 exhibited characteristic O‐O stretching band at 886 and 956 cm^−1^, which provides evidence for the direct 2e^−^ ORR pathway responsible for H_2_O_2_ generation (Figure 9f) [52]. Furthermore, as the reaction proceeded, the emergence of the C‐OH vibrational peak at 1092 cm^−^^1^ in both spectra of WYU‐HOF‐1 and WYU‐HOF‐2 demonstrated that adsorbed ^*^OH serves as a critical intermediate in the 2e^−^ indirect stepwise WOR process (Figure 9f; Figure S39) [51]. These findings directly confirmed the key intermediates involved in the H_2_O_2_ production process. Consequently, integrated results from the above quenching experiments, EPR spectroscopy, and RDE/RRDE analyses demonstrate that 2e^−^ ORR and 2e^−^ WOR proceed concurrently in both WYU‐HOF‐1 and WYU‐HOF‐2 systems, with distinct mechanistic pathways governing H_2_O_2_ generation: In WYU‐HOF‐1, H_2_O_2_ production occurs predominantly through an indirect two‐step 1e^−^ ORR mechanism, whereas WYU‐HOF‐2 facilitates dual reaction pathways, combining the indirect two‐step 1e^−^ ORR route with a direct single‐step 2e^−^ ORR process.

To further understand the mechanism of WYU‐HOF‐2 enhanced H_2_O_2_ production, contact angle measurements were carried out, which indicated that both WYU‐HOF‐1 and WYU‐HOF‐2 are hydrophilic (Figure S40). However, WYU‐HOF‐2 exhibits a slightly higher contact angle (32.0°) compared to WYU‐HOF‐1 (26.4°), which indicates that the introduction of C─F bonds creates a beneficial locally superhydrophobic interface in WYU‐HOF‐2 [46]. This interface effectively enhances the diffusion of H_2_O molecules and the mass transfer of O_2_, thereby optimizing the kinetics of ORR [53].

### 2.5 Theoretical Calculations

To identify the active sites and elucidate the distinct roles of WYU‐HOF‐1 and WYU‐HOF‐2 in the photocatalytic production of H_2_O_2_, DFT calculations were conducted to probe their capabilities in O_2_ adsorption and activation. Three electron‐rich sites were evaluated as potential ORR active centers. As displayed in Figures S41–S43, the O_2_ adsorption energies on WYU‐HOF‐2 are generally lower than those on WYU‐HOF‐1 across all evaluated sites. Notably, the acceptor benzyl (site B) exhibits the most substantial difference, with an adsorption energy of −1.99 eV for WYU‐HOF‐2 compared to −0.43 eV for WYU‐HOF‐1. This pronounced contrast suggests a preferential Yeager‐type O_2_ configuration on site B of WYU‐HOF‐2, and its significantly stronger adsorption implies superior thermodynamic favorability for O_2_ activation and subsequent reduction.

**FIGURE 10:**
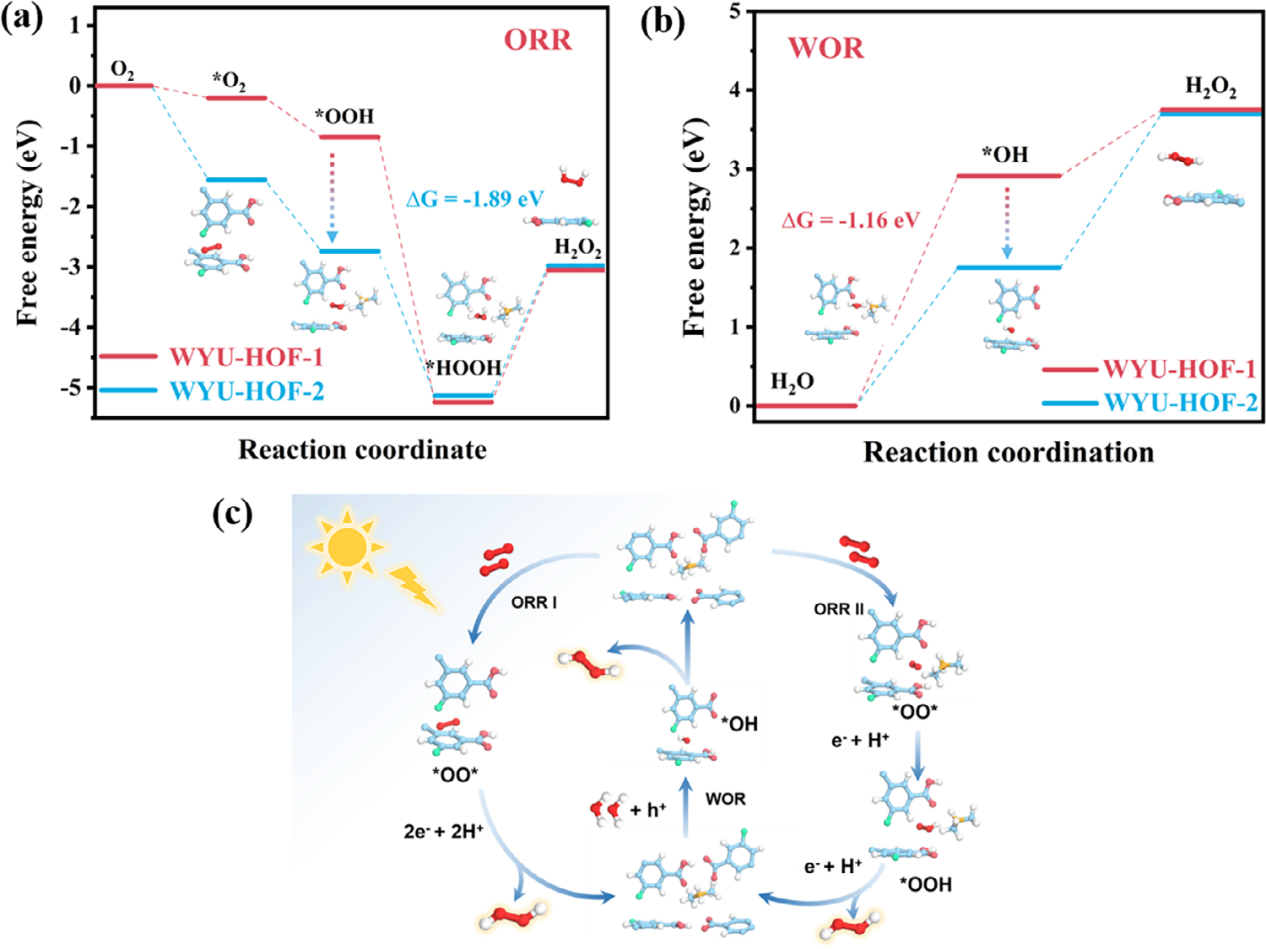
(a) Free‐energy diagrams for two‐step 1e^−^ ORR to H_2_O_2_ on the WYU‐HOF‐1 and WYU‐HOF‐2; (b) free‐energy diagrams for 2e^−^ WOR to H_2_O_2_ on the WYU‐HOF‐1 and WYU‐HOF‐2; (c) key intermediates and steps during the ORR and WOR pathways for H_2_O_2_ production on WYU‐HOF‐2.

Furthermore, the entire 2e^−^ ORR process on WYU‐HOF‐1 and WYU‐HOF‐2 was simulated to gain insight into the differences from a thermodynamic perspective. As shown in Figure 10a, the formation of ^*^OOH was identified as the step with the highest free energy cost among all elementary steps, establishing it as the rate‐determining step (RDS) for both materials. Crucially, the Gibbs free energy change (ΔG) for this step on WYU‐HOF‐2 was substantially lower (−2.74 eV) than that on WYU‐HOF‐1 (−0.85 eV). This significant difference confirms that the fluorine functionalization framework markedly facilitates O_2_ activation, rendering the ^*^OOH formation step more thermodynamically favorable. In the 2e^−^ WOR process, WYU‐HOF‐2 exhibits a reduced energy barrier of 1.75 eV for the dehydrogenation of H_2_O to ^*^OH, notably lower than the 2.91 eV required for WYU‐HOF‐1 (Figure 10b). This lower barrier indicates that the incorporation of fluorine functional groups significantly enhances the kinetics of H_2_O dehydrogenation.

To directly illustrate this mechanism, molecular dynamics (MD) simulations show the stepwise reduction of an O_2_ molecule to H_2_O_2_ (Movie S1). The process begins with the activation of O_2_ via hydrogen bonding with benzyl and HDMA^+^, forming an activated ^*^O_2_ species. This ^*^O_2_ then reacts with hydrogen species to form ^*^HOOH, which ultimately yields H_2_O_2_. Furthermore, simulations of O_2_ and H_2_O diffusion within the pore environment were conducted. The results show that the mean square displacement (MSD) of O_2_ was 1.67 × 10^−10^ m^2^ s^−^^1^, while that of H_2_O was 1.06 × 10^−10^ m^2^ s^−^^1^ (Figure S44). This indicates that O_2_ and H_2_O exhibit similar diffusion rates within WYU‐HOF‐2.

Based on the above theoretical calculations, a plausible photocatalytic mechanism has been proposed, using WYU‐HOF‐2 as an illustrative example (Figure 10c). H_2_O_2_ photosynthesis on WYU‐HOF‐2 proceeded via both the 2e^−^ ORR and the 2e^−^ WOR pathways. The ORR process mainly includes two pathways: direct one‐step 2e^−^ and indirect two‐step 1e^−^ pathways. In the direct pathway, O_2_ undergoes adsorption on the benzyl in a Yeager‐type conformation, followed by the simultaneous transfer of two protons (H^+^) and two electrons (e^−^) to form ^*^HOOH, ultimately leading to the generation of H_2_O_2_ (O_2_ + 2e^−^ + 2H^+^ → H_2_O_2_). In the indirect pathway, O_2_ is adsorbed on the benzyl in a Yeager‐type conformation, after which protons and electrons are transferred sequentially, resulting in the formation of various intermediates in a stepwise manner (O_2_ → •O_2_^−^ → ^*^OOH → ^*^HOOH → H_2_O_2_). In the 2e^−^ WOR pathway, the formation of ^*^OH constitutes the rate‐determining step (RDS). The overall conversion involves two key steps: first, H_2_O dehydrogenates to form ^*^OH; then, two ^*^OH intermediates combine to generate H_2_O_2_ (H_2_O→ •OH → H_2_O_2_).

## 3 Conclusions

In summary, a simple and effective strategy to engineer the number of charge transfer channels in hydrogen‐bonded organic frameworks (HOFs) by incorporating charge‐assisted hydrogen bonds has been developed. Furthermore, framework fluorination was found to create an additional channel. Crucially, the hydrogen bonds in HOFs could serve as atomic‐level pathways, which collectively significantly enhance the separation and migration efficiency of photoinduced charge carriers. Catalytic studies revealed that WYU‐HOF‐2, featuring three charge transfer pathways, exhibits a photocatalytic H_2_O_2_ production rate that is 3 times higher than that of WYU‐HOF‐1 (with two channels) and 62 times higher than that of FDU‐HOF‐3 (with one channel). Controlled experiments, RRDE measurements, in situ DRIFTS, and theoretical calculations collectively confirm that the photosynthetic generation of H_2_O_2_ proceeds via a dual‐pathway mechanism, involving both the 2e^−^ ORR and 2e^−^ WOR. Our work provides new insights into the rational design of HOFs by regulating charge transfer channels to boost the photocatalytic performance. Further work about the design and applications of the HOFs are in progress.

## 4 Experimental Section

### 4.1 Synthesis of WYU‐HOF‐1 Single Crystal

10 mg of H_4_PTTB was dissolved in 2 mL of DMF by ultrasonic treatment. Then 2 mL of solution was mixed with 1 mL of deionized water and 2 mL of HNO_3_ (2 mol/L) in a 20 mL vial. The mixture was kept in an oven at 100°C for 3 days. After cooling to room temperature, the yellow block‐shaped WYU‐HOF‐1 crystals were obtained. Anal Calcd for H_2_PTTB^2−^·2HDMA^+^ (C_48_H_40_N_2_O_8_): C,74.60; H, 5.21; N, 3.63%. Found: C,73.74; H, 4.93; N, 3.67%. FT‐IR (KBr) *ν* 3441 (w), 1689 (m), 1353 (s) cm^−1^.

### 4.2 Synthesis of WYU‐HOF‐2 Single Crystal

10 mg of H_4_PTTB‐F was dissolved in 2 mL of DMF by ultrasonic treatment; Then 2 mL of solution was mixed with 1 mL of deionized water and 2 mL of HNO_3_ (2 mol/L) in a 20 mL vial. The mixture stands at 100°C for 3 days. After cooling to room temperature, the yellow‐green needle‐like WYU‐HOF‐2 crystals were obtained. Anal Calcd for H_2_PTTB‐F^2−^·2HDMA^+^·2H_2_O (C_48_H_40_F_4_N_2_O_10_): C,65.45; H, 4.58; N, 3.18%. Found: C,65.15; H, 4.13; N, 3.87%. FT‐IR (KBr) *ν* 3441 (w), 1693 (m), 1383 (s), and 953 (m) cm^−1^.

[CCDC 2470449 and 2470106 contains the supplementary crystallographic data for this paper. These data can be obtained free of charge from The Cambridge Crystallographic Data Centre via www.ccdc.cam.ac.uk/data_request/cif.]

### 4.3 Photocatalytic H_2_O_2_ Production

10 mg of the photocatalyst was dispersed in 10 mL of deionized water. The reaction mixture in a 40 mL glass vial was bubbled with O_2_ for 30 min in the dark to achieve O_2_ saturation. The O_2_‐saturated suspension was then illuminated under a 300 W Xe lamp (λ > 400 nm) with magnetic stirring. The reaction temperature was maintained at room temperature throughout the experiment. After the reaction was complete, the solution was filtered through a 0.22 µm pore‐size membrane filter for H_2_O_2_ quantification. After measuring the absorbance of the mixture at 551 nm on a Shimadzu UV‐3600 spectrophotometer, the H_2_O_2_ concentration was determined based on a standard curve of H_2_O_2_ concentration‐absorbance.

### 4.4 Computational Details

First‐principles calculations for cell and geometry optimizations were performed using the QUICKSTEP module of the CP2K code (2023) [54], where the energies and geometry optimization were calculated using a hybrid Gaussian and plane‐wave (GPW) approach. The calculation employs the Perdew‐Burke‐Ernzerhof functional (GGA‐PBE) while the Kohn–Sham orbitals for all atoms are expanded into an atom‐centered double‐ζ quality DZVP‐MOLOPT‐GTH Gaussian basis set [55, 56]. The pseudopotentials used for all the atoms are those derived by Goedecker, Teter and Hutter. The cutoff energy of 600 Ry and the cutoff in reciprocal space of 60 are considered, respectively. The input file of CP2K was generated by Multiwfn software developed by Tian Lu [57]. Considering the calculation cost, the geometry optimization was only performed at Gamma point. The dynamic properties were modeled by using the Born–Oppenheimer molecular dynamics (BOMD) simulations as implemented in the CP2K package [58]. The simulations were sampled by the canonical (NVT) ensemble employing Nose–Hoover thermostats with a time step of 1.0 fs at a finite temperature of 300 K. The wave functions were expanded in optimized double‐ζ Gaussian basis sets, and the plane waves were expanded with a cutoff energy of 400 Ry.

## Conflicts of Interest

The authors declare no conflicts of interest.

## Supporting information




**Supporting File 1**: advs75201‐sup‐0001‐SuppMat.pdf.

**Supporting File 2**: advs75201‐sup‐0002‐Movid S1.mp4.

## Data Availability

The data that support the findings of this study are available from the corresponding author upon reasonable request.
