# Supplementary material for: Modulating the Charge Transfer Channels via Constructing Charge‐Assisted Hydrogen‐Bonded Organic Frameworks for Enhanced Photosynthesis of Hydrogen Peroxide
Source: Adv Sci (Weinh). 2026 Apr 7:e75201. Online ahead of print. doi: 10.1002/advs.75201 (PMC13334620; doi:10.1002/advs.75201)
Supplement: Supplementary file 1 — Supporting File 1: advs75201‐sup‐0001‐SuppMat.pdf. [file ADVS-9999-e75201-s002.pdf]

Modulating the Charge Transfer Channels via Constructing Charge-Assisted Hydrogen-Bonded Organic Frameworks for Enhanced Photosynthesis of Hydrogen Peroxide

Yajun Zhao, [a] Xianzhi Lan,[a] Tiantian Chen, [a] Qijie Mo, *[b] Chao Peng, [a] Jianbo Jia, [a] Jiewei Liu*[a], Cheng-Yong Su [c]

[a] School of Environmental and chemical Engineering, Jiangmen Key Laboratory of Synthetic Chemistry and Cleaner Production, Institute of Carbon Peaking and Carbon Neutralization, Wuyi University, Jiangmen 529020, P.R. China.

[b] School of Food & Pharmaceutical Engineering, Zhaoqing University, Zhaoqing, 526061, China.

[c] GBRCE for Functional Molecular Engineering, LIFM, IGCME, School of Chemistry, Sun Yat-Sen University, Guangzhou 510006, China

*Corresponding author: Dr. Q. Mo E-mail: sqjmo_jnu@163.com;

Dr. J. Liu E-mail: wyuchemliujw@126.com.

Table of Contents

1. General information....................S6
2. Experimental section....................S7
3. Ligand Synthesis....................S13
4. Crystal Structure and Characterizations of WYU-HOF-1 and WYU-HOF-2....................S16
5. Photoelectric Property....................S30
6. Photocatalytic H_2O_2 generation....................S33
7. Reaction meachanism....................S37
8. Theoretical calculation....................S43
9. NMR spectra of the Ligand....................S45
10. Reference....................S48

Captions for Figures and Tables

Scheme S1. Synthesis Procedure of H_4PTTB-R (R = H, F) Ligand.

Figure S1. Crystal photograph of WYU-HOF-1 and WYU-HOF-2.

Figure S2. Crystal structure of WYU-HOF-1.

Figure S3. Crystal structure of WYU-HOF-2.

Figure S4. The 4,4-connected sql topology with point symbol of $\{4^4{\cdot}6^2\}$ in FDU-HOF-3.

Figure S5. (a) Each H_2PTTB^{2-} units in WYU-HOF-1 is connected to four adjacent H_2PTTB^{2-} units and four adjacent $HDMA^+$ units; (b) 8-c node simplified from H_2PTTB^{2-} unit; (c) each $HDMA^+$ units is bridged by two H_2PTTB^{2-} units; (d) 2-c node simplified from $HDMA^+$ units; (d) the 2,8-connected topological net of WYU-HOF-1 with point symbol of $\{4^6{\cdot}6^{18}{\cdot}10^4\}\{4\}^2$.

Figure S6. (a) Each H_2PTTB-F2− units in WYU-HOF-2 is connected to four adjacent $H_2PTTB\text{-}F^{2-}$ units and four adjacent $HDMA^+$ units; (b) 8-c node simplified from $H_2PTTB\text{-}F^{2-}$ unit; (c) each $HDMA^+$ units is bridged by two $H_2PTTB\text{-}F^{2-}$ units; (d) 2-c node simplified from $HDMA^+$ units; (d) the 2,8-connected topological net of WYU-HOF-2 with point symbol of $\{4^6{\cdot}6^{18}{\cdot}10^4\}\{4\}^2$.

Figure S7. The FT-IR spectra of WYU-HOF-1 and WYU-HOF-2.

Figure S8. Experimental and simulated powder XRD patterns of WYU-HOF-1.

Figure S9. Experimental and simulated powder XRD patterns of WYU-HOF-2.

Figure S10. SEM and corresponding EDS mapping images of WYU-HOF-1.

Figure S11. SEM and corresponding EDS mapping images of WYU-HOF-2.

Figure S12. XPS survey of WYU-HOF-1 and WYU-HOF-2.

Figure S13. High-resolution XPS of C 1s in FDU-HOF-3, WYU-HOF-1 and WYU-HOF-2.

Figure S14. High-resolution XPS of O 1S in FDU-HOF-3, WYU-HOF-1 and WYU-HOF-2.

Figure S15. The TG curves of activated WYU-HOF-1 and WYU-HOF-2 samples.

Figure S16. Stability test of WYU-HOF-1 in different solvents for 7 days.

Figure S17. Stability test of WYU-HOF-1 in aqueous solutions with a pH range from 1 to 14 for 7

Figure S18. Stability test of WYU-HOF-2 in different solvents for 7 days.

Figure S19. Stability test of WYU-HOF-2 in aqueous solutions with a pH range from 1 to 14 for 7 days.

Figure S20. Electrostatic potential surface of H_4PTTB (left) and H_4-PTTB-F (right).

Figure S21. Mott–Schottky plot of WYU-HOF-1 in 0.1 M Na_2SO_4 aqueous solution (PH 6.5).

Figure S22. Mott–Schottky plot of WYU-HOF-2 in 0.1 M Na_2SO_4 aqueous solution (pH 6.5).

Figure S23. Standard curve for H_2O_2 detection.

Figure S24. UV–vis diffuse reflection spectroscopy (UV/Vis DRS) spectrum and the apparent quantum yield (AQY) of WYU-HOF-2 for H_2O_2 generation.

Figure S25. Photocatalytic H_2O_2 yield by WYU-HOF-2 in pure water, tap water, lake water and river water under visible-light irradiation.

Figure S26. Recycle experiment

Figure S27. The SEM images of WYU-HOF-1 after photocatalytic reaction.

Figure S28. The SEM images of WYU-HOF-2 after photocatalytic reaction.

Figure S29. The PXRD patterns of WYU-HOF-1 before and after photocatalysis

Figure S30. The PXRD patterns of WYU-HOF-2 before and after photocatalysis.

Figure S31. Controlled experiment of WYU-HOF-2 for H_2O_2 photosynthesis.

Figure S32. The amount of H_2O_2 generated over WYU-HOF-2 in the presence of different scavengers under irradiation for 1 h (p-BQ 5 mM; DMPO 4% V/V).

Figure S33. EPR spectra of DMPO-•OH for WYU-HOF-1 and WYU-HOF-2.

Figure S34. Linear-sweep RDE voltammograms of WYU-HOF-1 measured at different rotating speeds in phosphate buffer solution (pH = 7) with continuous O_2 purging.

Figure S35. Linear-sweep RDE voltammograms of WYU-HOF-2 measured at different rotating speeds in phosphate buffer solution (pH = 7) with continuous O_2 purging.

Figure S36. RRDE voltammograms of WYU-HOF-2 obtained in phosphate buffer (pH = 7) at rotation speed of 1600 rpm. The potential of Pt ring electrode was set at +0.23 V vs. Ag/AgCl to detect O_2.

Figure S37. RRDE voltammograms of WYU-HOF-1 obtained in phosphate buffer (pH = 7) at rotation speed of 1600 rpm. The potential of Pt ring electrode was set at +0.23 V vs. Ag/AgCl to detect O_2.

Figure S38. RRDE voltammograms of WYU-HOF-1 obtained in phosphate buffer (pH = 7) at rotation speed of 1600 rpm. The potential of Pt ring electrode was set at +0.6 V vs. Ag/AgCl to detect H_2O_2.

Figure S39. DRIFTS spectra of WYU-HOF-1 under the saturated O_2 condition in pure water.

Figure S40. Water contact angle measurements for (a) WYU-HOF-1 and (b) WYU-HOF-2.

Figure S41. Different O_2 adsorption configurations and adsorption energy for WYU-HOF-1.

Figure S42. Different O_2 adsorption configurations and adsorption energy for WYU-HOF-2.

Figure S43. The adsorption energy of O_2 for WYU-HOF-1 and WYU-HOF-2.

Figure S44. MSD of O_2 and H_2O diffusion in WYU-HOF-2.

Table S1. Summary of crystallographic data and refinement results of WYU-HOF-1 and WYU-HOF-2.

Table S2. Selected bond lengths (Å) and angles (º) of WYU-HOF-1.

Table S3. Selected bond lengths (Å) and angles (º) of WYU-HOF-2.

1. General information

All chemicals used in this work were purchased from commercial supplies without further purification. Powder X-ray diffraction (PXRD) studies were carried out on a Rigaku MiniFlex 600-C diffractometer (Bragg-Brentano geometry, Cu-Ka radiation, λ= 1.54178 Å).). ^{1}H was recorded on Bruker AVANCE III 500(500 MHz).The morphologies of the samples were observed using scanning electron microscopy (SEM, Zeiss Gemini SEM 500 apparatus) with an energy-dispersive X-ray spectrometry (EDS). Samples for SEM tests were dispersed in EtOH with the aid of sonication, and then deposited on a conductive tape. Fourier transform infrared (FT-IR) spectra were obtained with KBr pellets using a Bruker Tensor 27 FT-IR spectrometer. XPS analyses were performed on a Thermo Scientific ESCALAB 250Xi with a monochromatized micro-focused Al Ka X-ray source provided by eceshi (www.eceshi.com). The powder samples were pressed onto double-sided adhesive tape and mounted on the sample holder. No Ar^{+} sputtering was performed prior to analysis. Binding energies (BE) were calibrated by setting the measured BE of C Is to 284.65 eV. UV-vis absorption spectra were recorded on a Shimadzu UV-3600 Plus spectrometer. Fluorescence spectra were measured on an Edinburgh FLS1000 Photoluminescence Spectrometer. The fluorescence lifetime experiments were performed in the time-correlated single photo counting (TCSPC) methods by using the picoseconds pulsed diode laser. Femtosecond time-resolved transient absorption (TA) measurements were carried out on a Ultrafast Systems LLC spectroscopy.

2. Experimental Section

2.1 Single Crystal X-ray Crystallography

The X-ray diffraction data was collected with a Rigaku Super Nova X-RAY diffractometer system equipped with Cu-kα radiation (λ = 1.54178 Å). The crystal was kept at 298 K during data collection. The structure was solved with the SIR2004 structure solution program integrated in Olex2 using Direct Methods, and refined with the XH refinement package using CGLS minimization.[1] The structure was solved with the ShelXS structure solution program integrated in Olex2 using Direct Methods, and refined with the ShelXL refinement package using CGLS minimization. The positions of the hydrogen atoms are generated geometrically. A summary of the crystal structure refinement data and selected bond angles and distances are provided in Tables S1-S3. Crystallographic data for the structure have been deposited in the Cambridge Crystallographic Data Center with CCDC reference number 2470449 (WYU-HOF-1) and 2470106 (WYU-HOF-2).

2.2 Photoelectrochemical characterization

Photoelectrochemical measurements were performed on a CHI 660E electrochemical work station (Chenhua Instrument, Shanghai, China) in a standard three-electrode system with the photocatalyst-coated FTO, Pt plate and Ag/AgCl as the working electrode, counter electrode and reference electrode, respectively. The as-synthesized samples (4 mg) were added into Nafion (20 μL) and acetone (180 μL) mixed solution, giving a suspension, and then working electrodes were prepared by dropping the suspension (40 μL) onto the surface of a FTO plate. The working electrodes were dried at room temperature. The photocurrent was measured using constant voltage tracking (CVT) using a 0.1 M Na_2SO_4 solution as the electrolyte. A 300 W Xe lamp ($\lambda \geq 420$ nm) was used as the light source, and a shutter was used to modulate the light and dark conditions during the test. Photo-responsive signals of the samples were measured under chopped light at 0.5 V. The electrochemical impedance spectroscopy (EIS) was performed in frequency range from 10^{-2} to 10^{2} Hz with a bias potential of 1.5 V. The Mott-Schottky measurements were performed at frequencies of 500, 1000, and 1500 Hz, respectively.

2.3 Photocatalytic H_2O_2 production

To investigate the influence of different sacrificial agents on the H_2O_2 generation activity, 5,5-dimethylpyrrolidine N-oxide (DMPO, 4%, V/V), tert-butyl alcohol (TBA, 4%, V/V), $AgNO_3$ (2 mM) and Methanol (MeOH, 4%, V/V) were added to the reaction system separately. To explore the influence of N_2 on the photocatalytic H_2O_2 generation activity, N_2 was continuously injected into the reaction solution under dark conditions for 30 min.

2.4 H_2O_2 detection method

The concentration of H_2O_2 was determined using the N, N-diethyl-1,4-phenyl diamine sulphate (DPD) colorimetric method.[2] The DPD stock solution was prepared by dissolving 100 mg of DPD in 10 mL of 0.05 M H_2SO_4 with thorough stirring. The phosphate buffer solution was obtained by mixing 87.7 mL of 1.0 M NaH_2PO_4, 12.6 mL of 1.0 M Na_2HPO_4, and 99.7 mL of DI water. Peroxidase (POD, horseradish) solution was freshly prepared by dissolving 5.0 mg of POD in 5 mL of deionized water and stored in a refrigerator prior to use. During experiments, the reaction suspension was filtered through a 0.22 μm PTFE filter. Then 1 mL aliquot of the filtered suspension was mixed with 3 mL of phosphate buffer, 50 μL of DPD solution, and 50 μL of POD solution. The mixture was diluted to 10 mL and shaken for 90 s to ensure homogeneity. The absorbance was measured at 551 nm using a UV/Vis spectrophotometer. Standard solutions ranging from 25 to 300 μM were prepared by diluting a 0.1% H_2O_2 stock solution, and a calibration curve was constructed for quantitative analysis of H_2O_2 in the samples (Figure S23).

2.5 H_2O_2 decomposition experiment

A decomposition experiment of H_2O_2 was conducted with catalysts (10 mg) in an aqueous solution (10 mL) containing H_2O_2 (1 mM) under N_2 atmosphere. A 300 W Xe lamp (PLS-SXE300, Beijing Perfect Light) was used as the source. The H_2O_2 residue was measured.

2.6 Electron paramagnetic resonance (EPR) measurements

Hydroxyl radical (•OH) detection: The spectra were collected from a Bruker EMXnano spectrometer at room temperature using 5,5-dimethyl-1-pyrroline-N-oxide (DMPO) as the trapping agent. 3 mg HOF sample was dispersed in aqueous solution containing 1 mL CH_3OH and 60 μL trapping agent. The whole mixture was irradiated for 300 s under visible light ($\lambda \geq 420$ nm) with vigorously stirring. After that, 200 μL of the suspension was injected into a glass capillary and placed in a glass tube in an air environment. The glass tube was placed in the microwave cavity of EPR spectrometer for analysis.

Superoxide radical (•O_2^-) detection: The spectra were collected from a Bruker EMXnano spectrometer at room temperature using 5,5-dimethyl-1-pyrroline-N-oxide (DMPO) as the trapping agent. 3 mg HOF sample was dispersed in aqueous solution containing 1 mL CH_3OH and 60 μL trapping agent. The whole mixture was irradiated for 300 s under visible light ($\lambda \geq 420$ nm) with vigorously stirring. After that, 200 μL of the suspension was injected into a glass capillary and placed in a glass tube in an air environment. The glass tube was placed in the microwave cavity of EPR spectrometer for analysis.

2.7 In situ irradiated X-ray photoelectron spectroscopy (ISI-XPS)

In situ irradiated X-ray photoelectron spectroscopy (XPS) measurements were conducted on a Thermo Scientific ESCALAB 250Xi with a monochromatized micro-focused Al Ka X-ray source. All binding energies were referenced to the adventitious C 1s line at 284.65 eV. The powder samples were pressed onto double-sided adhesive tape and mounted on the sample holder. No Ar^+ sputtering was performed prior to analysis. A 300 W Xe lamp ($\lambda \geq 400$ nm) (Perfect Light) was kept ≈ 15 cm away from the samples as a light source.

2.8 In-situ DRIFTS measurements

DRIFTS measurements were performed on a Nicolet iS50 FT-IR. The photocatalyst was filled into an in-situ IR holder in the chamber. Before the measurement, the chamber was degassed under argon flow at a rate of 30 mL min^{-1} for 30 min. The baseline was then obtained at room temperature. Then, oxygen and water vapor were purged into the chamber for 30 min under dark condition. Followed by irradiation under visible light (λ > 420 nm) through the window of the chamber, the in-situ FTIR spectra were recorded at specific intervals.

2.9 Measurement of apparent quantum yield

The measurement of apparent quantum yield (AQY) was measured under the illumination of a 300 W Xe lamp (PLSSXE300, Beijing Perfect Light) with different bandpass of 420, 500, 600 nm. After ultrasonication and air bubbling, the photocatalytic reaction was carried out in deionized water (10 mL) with photocatalyst (10 mg). The active area of the reactor was approximately 2 cm^2. The monochromatic light intensity was averaged at three representative points with CEL-NP2000 Beijing Ceaulight Photo radiometer. Therefore, the calculated light intensity at 420 nm was 3.3 mW cm^{-2}. AQY was calculated by the following formula:

$$\mathrm{AQY} = \frac{(\text{number of } \mathrm{H_2O_2} \text{ production}) \times 2}{\text{number of incident photos}} \times 100\%$$

The number of incident photons is:

$$\mathrm{N_{incident}} = \frac{\mathrm{Pt}}{\mathrm{h\nu}} = \frac{\mathrm{Pt\lambda}}{\mathrm{hc}} = \frac{\mathrm{ISt\lambda}}{\mathrm{hc}}$$

where I = light powder intensity (W cm^{-2}); S = the irradiation area (cm^2); t = reaction time (s); λ = wavelength (nm); h = 6.63 x 10-^{34} m^2 Kg s^{-1} (Planck's constant); c = 3 x 10^8 s^{-1} (speed of light)

2.10 Rotating disk electrode (RDE) measurements.

A rotating disk electrode (PINE Corporation, USA) was served as the substrate for the working electrode. To prepare the catalyst ink, 5 mg of catalysts were dispersed in a mixture of 490 μL ethanol and 10 μL Nafion solution (2 wt%) and then subjected to ultrasonic treatment to ensure homogeneity. A volume of 20 μL of the slurry was placed on the disk electrode and dried at room temperature. The linear sweep voltammograms (LSV) was recorded on an MSRX electrode rotator (Pine Instrument) and the CHI 760E workstation (Chenhua Instruments, China), with a Pt wire and Hg/HgO electrodes serving as the counter and reference electrodes, respectively. The LSV were obtained in an O_2-saturated 0.1 M phosphate buffer solution (PBS, pH = 7) solution at room temperature with a scan rate of 5 mV s^{-1} and different rotation speeds. The average number of electrons (n) was calculated by the Koutecky-Levich equation:

$$\frac{1}{J}=\frac{1}{J_L}+\frac{1}{J_K}=\frac{1}{B\omega_{1/2}}+\frac{1}{J_K} \quad (1)$$

$$B=0.62\ n\ F\ C_0\ D_0^{\ 2/3}\ v^{-1/6} \quad (2)$$

Where J is the current density, J_K and J_L are the kinetic and diffusion-limiting current densities, ω is the rotating speed (rpm), n is the transferred electron number, F is Faraday constant (96485 C mol^{-1}), C_0 is the bulk concentration of O_2 (1.26 $\times 10^{-3}$ mol cm^{-3}), D_0 is the diffusion coefficient of O_2 (2.7 $\times 10^{-5}$ cm^2 s^{-1}), and ν is kinetic viscosity of the electrolyte (0.01 cm^2 s^{-1}), respectively.

2.11 Rotating ring-disk electrode (RRDE) measurement

A ring-disk electrode (PINE Research Instrumentation, USA) was served as the substrate for working electrode. RRDE measurements were carried on the CHI842B workstation (CH Instruments, Inc.) with a RRDE-3A rotator (ALS Co., Ltd). A Pt wire and Ag/AgCl electrodes were used as counter electrode and reference electrodes, respectively. For the electrode modification, 6 mg of catalyst powder was dispersed in a mixture (3.0 mL) of water, isopropyl alcohol, and Nafion with a volume ratio of 20:1:0.075. Then the mixture was ultrasonicated for 2 h to generate a homogeneous ink, which was then transferred onto the electrode surface with a loading mass of 1000 μg cm^{-2}. The voltammograms were obtained in a 0.1 M phosphate buffer solution (pH = 7) at room temperature under N_2 atmosphere with a scan rate 5 mV s^{-1} and a rotation rate of 1600 rpm. The phosphate buffer solution electrolyte was purged with N_2 for 30 min prior to the measurements.

The potential of ring electrode was set to -0.23V and 0.6 V (vs. Ag/AgCl) to detect O_2 or H_2O_2, respectively.

3. Ligand Synthesis

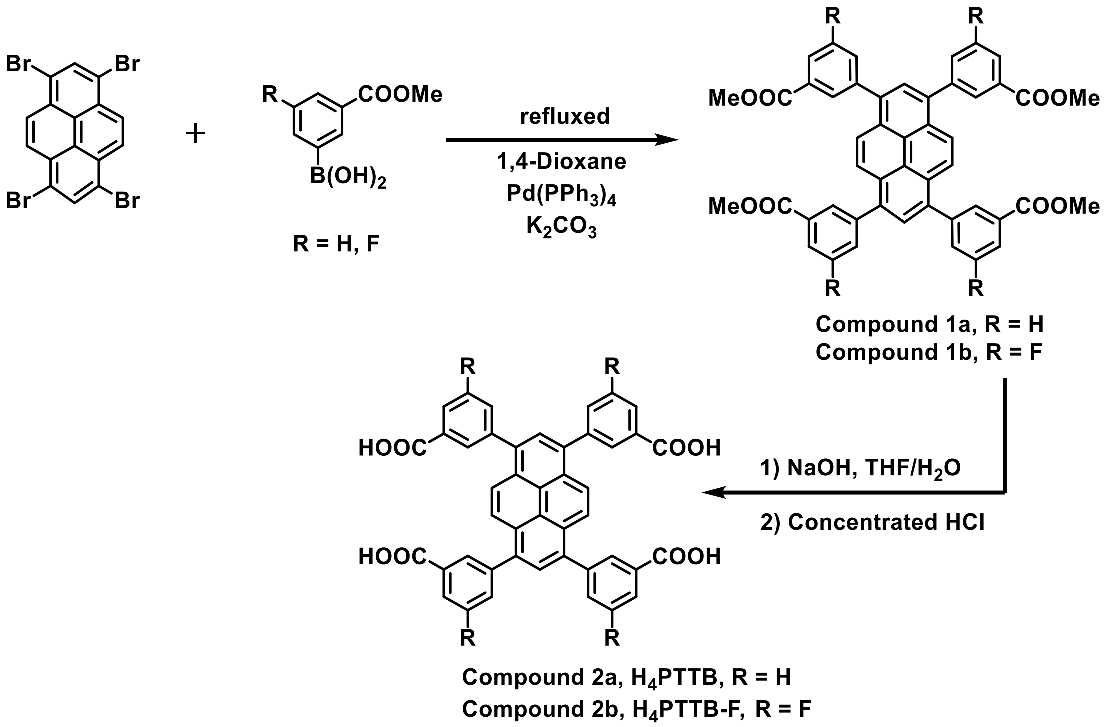

Scheme S1. Synthesis Procedure of H_4PTTB-R (R = H, F) Ligand

(1) 1,3,6,8-tetrakis(3-(methoxycarbonyl)phenyl)pyrene (1a).[3] A mixture of 1,3,6,8-tetrabromopyrene (4.26 g, 8.25 mmol), (3-(methoxycarbonyl) phenyl)boronic acid (8.9 g, 49.5 mmol), potassium carbonate (9.0 g, 66 mmol), palladium tetrakis- (triphenylphosphine) (0.2 g, 0.18 mmol) in dry dioxane (120 mL) was stirred under argon for 72 h at 100°C in an oil bath. The reaction mixture was evaporated to dryness and the obtained solid residue was washed with 1 M HCl solution, followed by water to remove the inorganic salts. The insoluble material was extracted with chloroform (40 mL x 4), the organic phases were combined. To the organic phases, 1000 mL methanol was added, then a light-yellow solid precipitate was formed, finally the solid was collected by filtration, washed with methanol, giving 5.6 g of 1,3,6,8-tetrakis(3-(methoxycarbonyl) phenyl)pyrene (93% yield). ^{1}H NMR (500 MHz, $CDCl_3$) δ 8.37 (s, 4H), 8.19 (d, J = 7.9 Hz, 4H), 8.14 (s, 4H), 8.04 (s, 2H), 7.89 (d, J = 7.7 Hz, 4H), 7.66 (t, J = 7.7 Hz, 4H), 3.97 (s, 12H).

(2) 1,3,6,8-tetrakis(3-carboxyphenyl)pyrene (H_4PTTB).[3] To a 500 mL round bottom flask containing 2.8 g (3.8 mmol) of solid 1,3,6,8-tetrakis(3-(methoxycarbonyl)-phenyl) pyrene, a

solution containing 10 g (250 mmol) NaOH in 320 mL of a THF/water (ratio 1:1) mixture was added and the resultant suspension was vigorously stirred under reflux for 48 h. The solvents were removed under vacuum and water was added to the residue, which formed a clear yellow solution. Then the homogeneous solution was acidified with concentrated HCl until no further precipitate was detected (pH ~ 1). The yellow solid was collected by filtration, washed with water and methanol, and dried in vacuum. Yield: 2.5 g, 96%. ^{1}H NMR (500 MHz, DMSO-d_6) δ 13.14 (s, 4H), 8.24 (s, 4H), 8.17 (s, 4H), 8.11 (t, J = 6.2 Hz, 4H), 8.08 (s, 2H), 8.00 (d, J = 7.8 Hz, 4H), 7.75 (t, J = 7.7 Hz, 4H).

(3) 1,3,6,8-tetrakis(3-fluoro-5-(methoxycarbonyl) phenyl) pyrene (1b). A mixture of 1,3,6,8 tetrabromopyrene (0.71 g, 1.325 mmol), 3-fluoro-(5-(methoxycarbonyl) phenyl) boronic acid (1.63 g, 8.25 mmol), potassium carbonate (1.5 g, 10.95 mmol), palladium tetrakis- (triphenylphosphine) (0.03 g, 0.027 mmol) in dry dioxane (40 mL) was stirred under argon for 72 h at 100 °C in an oil bath. After cooling to room temperature, the solvent was removed under reduced pressure. The solid was then dissolved in water, and concentrated hydrochloric acid was added dropwise until no further gas evolution was observed. The resulting yellow-green suspension was extracted with chloroform. The combined organic layers were concentrated under reduced pressure to afford a yellow-green solid. This solid was then dissolved in acetone, forming a suspension, which was filtered under vacuum to afford 1.2 g of pale yellow-green 1,3,6,8-tetrakis(3-fluoro-5-(methoxycarbonyl) phenyl) pyrene solid (91% yield). ^{1}H NMR (400 MHz, $CDCl_3$) δ 8.14 (s, 8H), 8.00 (s, 2H), 7.86 (d, J = 8.6 Hz, 4H), 7.58 (d, J = 8.8 Hz, 4H), 3.96 (s, 12H).

(4) 1,3,6,8-tetrakis(3-fluoro-5-carboxyphenyl) pyrene (H_4PTTB-F). To a 500 mL round bottom flask containing 1.2 g of solid 1,3,6,8-tetrakis(3-fluoro-5-(methoxycarbonyl) phenyl) pyrene, a solution containing 3 g (75 mmol) NaOH in 320 mL of a THF/water (ratio 1:1) mixture was added and the resultant suspension was vigorously stirred under reflux for 48 h. The solvents were removed under vacuum and water was added to the residue, which formed a clear yellow solution. Then the homogeneous solution was acidified with concentrated HCl until no further precipitate was detected (pH ~ 1). The yellow-green solid was collected by filtration, washed with water and methanol, and

dried in vacuum. Yield: 1 g, 97% yield.^{1}H NMR (500 MHz, DMSO-d_6) δ 13.48 (s, 4H), 8.15 (s, 4H), 8.09 – 8.02 (m, 6H), 7.91 (dt, *J* = 9.3, 2.2 Hz, 4H), 7.81 (ddd, *J* = 9.1, 2.6, 1.3 Hz, 4H).

Synthesis of powder WYU-HOF-1 with microcrystalline. 200 mg of H_4PTTB was dissolved in 10 mL of DMF by ultrasonic treatment. Then 10 mL of solution was mixed with 5 mL of deionized water and 10 mL of HNO_3 (2 mol/L) in a 40 mL vial. The mixture stands at 100 °C in an oven for 3 days. After cooling to room temperature, the powder was washed with DMF and acetone for three times, respectively. The final product was dried in a vacuum at 60 °C for 6 h to obtain the yellow-green powder (190 mg, 87% yield based on H_4PTTB)

Synthesis of powder WYU-HOF-2 with microcrystalline. 200 mg of H_4PTTB-F was dissolved in 10 mL of DMF by ultrasonic treatment. Then 10 mL of solution was mixed with 5 mL of deionized water and 10 mL of HNO_3 (2 mol/L) in a 40 mL vial. The mixture stands at 100 °C in an oven for 3 days. After cooling to room temperature, the powder was washed with DMF and acetone for three times, respectively. The final product was dried in a vacuum at 60 °C for 6 h to obtain the yellow-green powder (192 mg, 88% yield based on H_4PTTB-F)

Synthesis of powdery FDU-HOF-3 with microcrystalline. FDU-HOF-3 was synthesized according to the previous literature.[4] Typically, H_4PTTB (200 mg, 0.292 mmol) was dissolved in 10 mL of NMP in a 40 mL uncapped vial. The vial was put in a 100 mL vial containing 30 mL of methanol. The mixture stands at 80°C for 12 hours to afford yellow block crystals of FDU-HOF-3 (170 mg, Yield: 85%)

4. Crystal Structure and Characterizations of WYU-HOF-1 and WYU-HOF-2

Table S1. Summary of crystallographic data and refinement results of WYU-HOF-1 and WYU-HOF-2.

**Compounds**	WYU-HOF-1	WYU-HOF-2
Formula	$C_{48}H_{40}N_2O_8$	$C_{48}H_{36}F_4N_2O_8$
Fw	772.82	844.79
T/K	297	150.15
Crystal system	monoclinic	monoclinic
Space group	I2/a	C2/c
a/Å	8.0424(1)	31.1105(7)
b/Å	15.0378(2)	15.3739(4)
c/Å	31.9327(5)	8.1097(2)
α/°	90	90
β/°	94.139(2)	95.002(2)
γ/°	90	90
Volume/Å^3	3851.87(9)	3864.01(16)
Z	4	4
ρ_{calc}g/cm^3	1.329	1.445
μ/mm^{-1}	0.739	0.943
F(000)	1616	1736.0
Reflections collected	26578	13100
Independent reflections	4021	3842
Data/restraints/parameters	4021/1/270	3842/60/285
R_{int}	0.0533	0.0405
Goodness-of-fit on F^2	1.031	1.149
R1, w*R2* [I>=2σ (I)]	0.0534, 0.1472	0.1041, 0.2127
R1, w*R2* [all data]	0.0600, 0.1531	0.1189, 0.2188

Table S2. Selected bond lengths (Å) and angles (º) of WYU-HOF-1

**Bond Length (Å)**		**Bond Angle (º)**			
O2-C1	1.239(2)	C10[1]-C11-C10	120.21(18)	C16[1]-C17-C16	122.82(19)
O4-C24	1.289(2)	C10[1]-C11-C12	119.89(9)	C7-C2-C1	118.77(14)
O3-C24	1.217(2)	C10-C11-C12	119.89(9)	C7-C2-C3	119.48(14)
O1-C1	1.270(2)	C11-C10-C15	117.81(13)	C3-C2-C1	121.73(14)
C11-C10	1.4279(16)	C8-C10-C11	119.13(13)	O4-C24-C22	115.36(15)
C11-C101	1.4279(16)	C8-C10-C15	123.00(12)	O3-C24-O4	123.91(16)
C11-C12	1.433(3)	C10-C8-C6	123.12(12)	O3-C24-C22	120.72(15)
C10-C8	1.414(2)	C9-C8-C10	118.55(13)	O2-C1-O1	124.02(16)
C10-C15	1.4346(19)	C9-C8-C6	118.27(14)	O2-C1-C2	119.09(14)
C8-C9	1.3890(17)	C12-C13-C14	117.97(14)	O1-C1-C2	116.85(16)
C8-C6	1.4872(18)	C16-C13-C12	119.36(13)	C18-C19-C20	120.88(15)
C16-C18	1.4961(19)	C13-C12-C131	119.87(18)	C21-C20-C19	120.46(16)
C16-C17	1.3912(17)	C8-C9-C81	123.60(19)	C26-N1-C25	112.8(3)
C14-C15	1.346(2)	C13-C16-C18	121.68(12)		
C4-C3	1.379(2)	C23-C22-C24	118.52(14)		
C21-C20	1.384(3)	C21-C22-C23	119.74(14)		
N1-C26	1.446(5)	C21-C22-C24	121.74(14)		
N1-C25	1.480(5)	C4-C5-C6	120.92(14)		

Table S3. Selected bond lengths (Å) and angles (º) of WYU-HOF-2

Bond Length (Å)		Bond Angle (º)			
O1-C1	1.294(10)	C13-C16-C18	123.5(5)	C18-C21-C22	121.9(6)
C16-C13	1.417(8)	C17-C16-C13	118.9(5)	C23-C20-C19	123.2(6)
C16-C17	1.386(7)	C17-C16-C18	117.6(5)	F2-C20-C19	118.7(6)
C16-C18	1.487(7)	C16-C13-C15	122.5(5)	F2-C20-C23	118.1(6)
C13-C15	1.435(8)	C16-C13-C12	118.7(5)	C3-C2-C7	118.0(6)
C14-C10	1.440(8)	C15-C14-C10	121.2(5)	C6-C7-C1	122.5(7)
C14-C15	1.348(8)	C81-C9-C8	122.6(7)	C2-C7-C6	119.9(6)
C11-C101	1.419(7)	C10^{1}-C11-C10	120.4(7)	C2-C7-C1	117.7(6)
C11-C10	1.420(7)	C10-C11-C12	119.8(4)	O3-C24-C22	118.6(6)
C11-C12	1.443(11)	C10^{1}-C11-C12	119.8(4)	O4-C24-O3	124.0(7)
O3-C24	1.256(10)	C8-C10-C14	121.9(5)	O4-C24-C22	117.3(7)
C18-C19	1.397(8)	C8-C10-C11	119.4(5)	O1-C1-C7	114.6(7)
C18-C21	1.392(8)	C11-C10-C14	118.6(5)	O2-C1-O1	124.1(7)
C5-C6	1.383(8)	C14-C15-C13	121.7(5)	O2-C1-C7	121.3(8)
C4-C3	1.373(9)	C16-C17-C161	123.1(7)	C25-N1-C26	108.9(13)
C19-C20	1.376(8)	C19-C18-C16	120.0(5)		
O2-C1	1.220(11)	C2-C3-C4	122.9(6)		
N1-C26	1.424(15)	F1-C3-C4	118.0(6)		
N1-C25	1.407(18)	F1-C3-C2	119.2(6)		

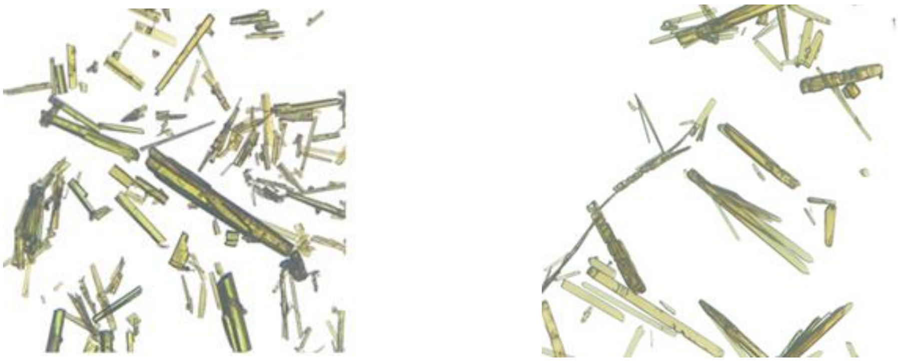

Figure S1. Crystal photograph of WYU-HOF-1 and WYU-HOF-2.

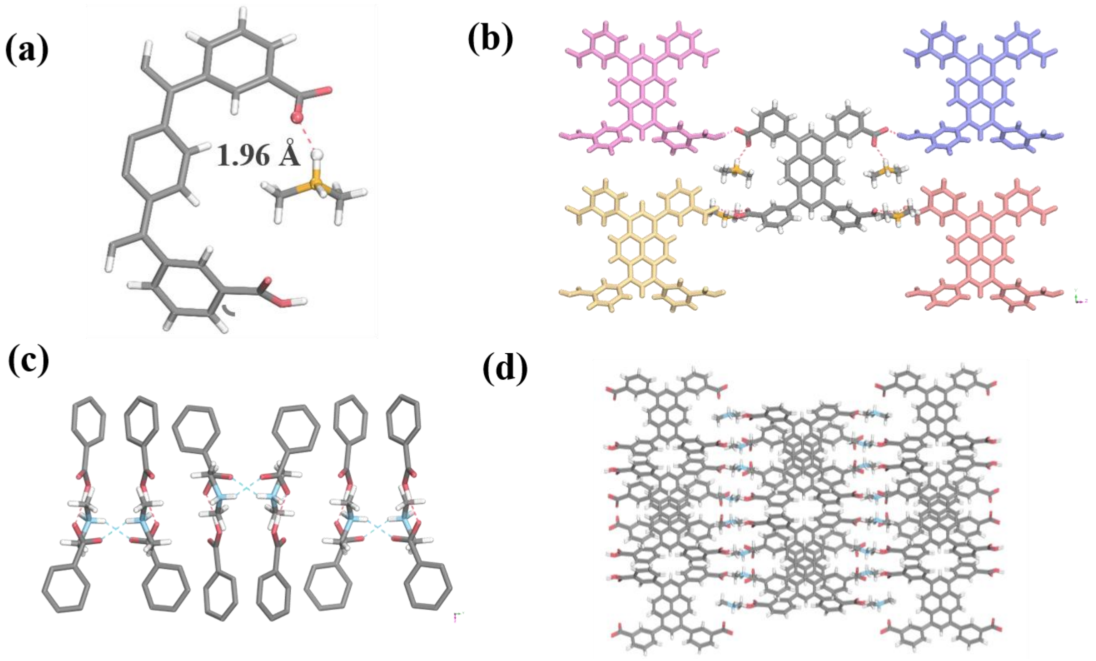

Figure S2. Crystal structure of WYU-HOF-1. (a) The asymmetric unit; (b) the coordination mode of H_2PTTB^{2-} units with $HDMA^+$ cation; (c) H-bonded chain, the adjacent rhombic networks in WYU-HOF-1 are connected by $HDMA^+$ through N–H···O hydrogen bonds; (d) 3D packing mode in the structure of WYU-HOF-1. Color codes: N, yellow; O, red; C, gray; H, white.

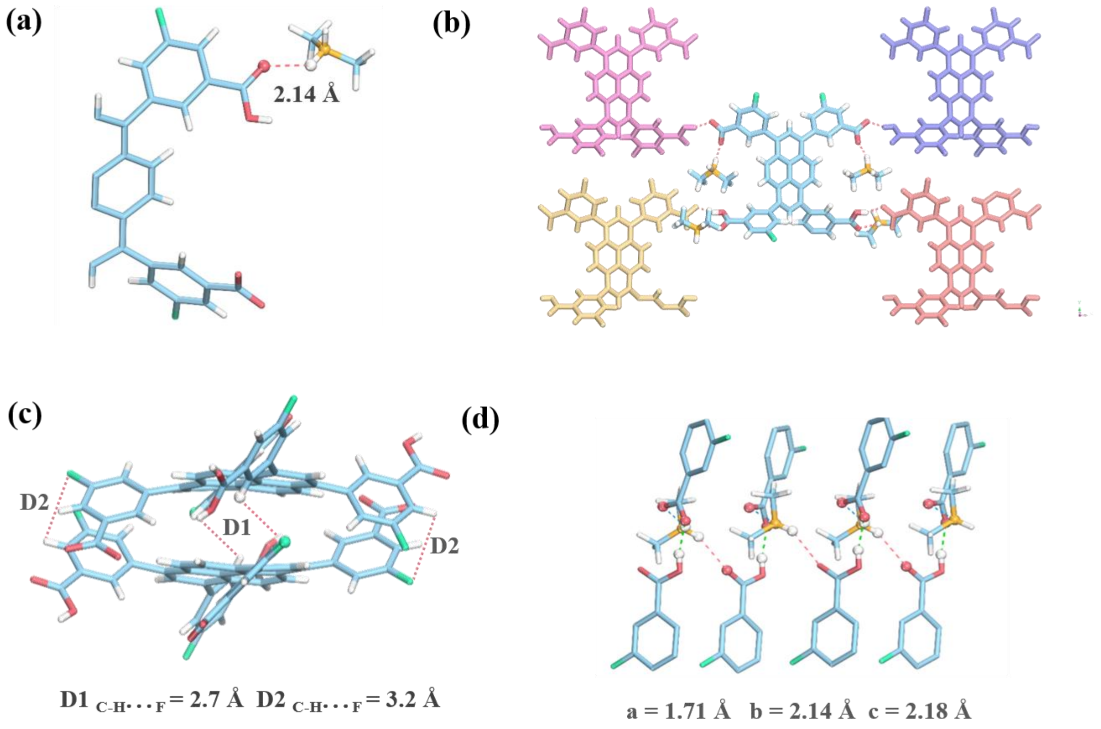

Figure S3. Crystal structure of WYU-HOF-2. (a) The asymmetric unit; (b) the coordination mode of H_2PTTB-F^{2-} units: the H_2PTTB^{2-} unit was connected with four neighboring H_2PTTB-F^{2-} and four $HDMA^+$ through hydrogen bonds; (c) π–π stacking interactions and other weak interaction in WYU-HOF-2. (d) H-bonded chain, the adjacent rhombic networks in WYU-HOF-2 are connected by $HDMA^+$ through N–H···O hydrogen bonds. Color codes: N, yellow; F, green; O, red; C, blue; H, white.

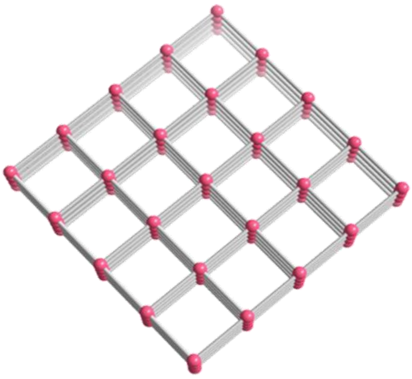

Figure S4. The 4,4-connected **sql** topology with point symbol of $\{4^4{\cdot}6^2\}$ in FDU-HOF-3.

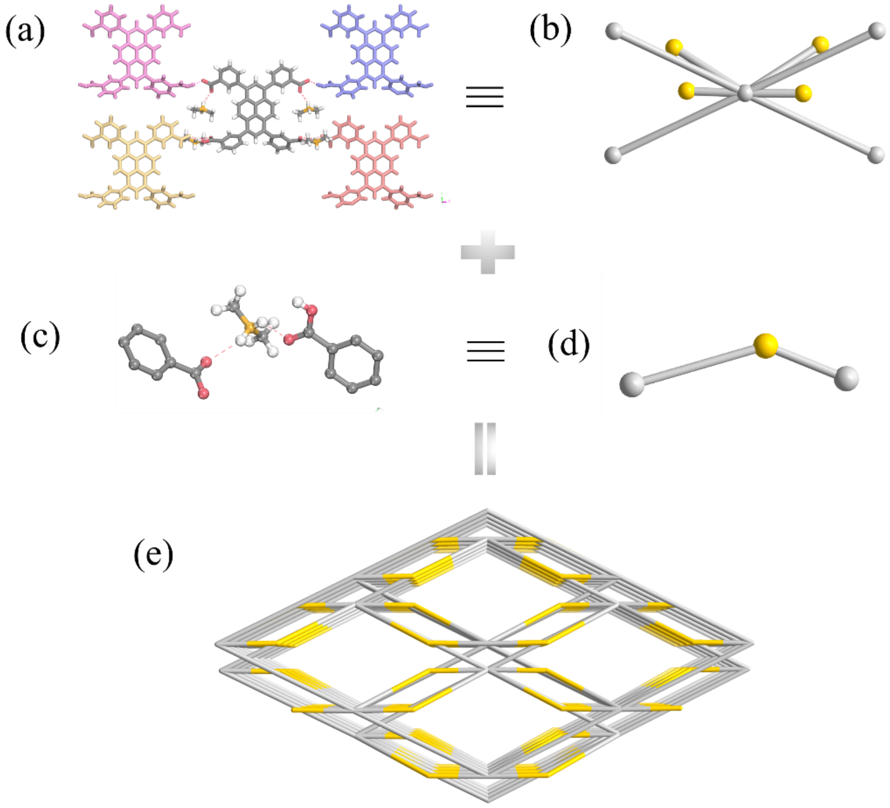

Figure S5. (a) Each H_2PTTB^{2-} units in WYU-HOF-1 is connected to four adjacent H_2PTTB^{2-} units and four adjacent $HDMA^+$ units; (b) 8-c node simplified from H_2PTTB^{2-} unit; (c) each $HDMA^+$ units is bridged by two H_2PTTB^{2-} units; (d) 2-c node simplified from $HDMA^+$ units; (d) the 2,8-connected topological net of WYU-HOF-1 with point symbol of $\{4^6{\cdot}6^{18}{\cdot}10^4\}\{4\}^2$.

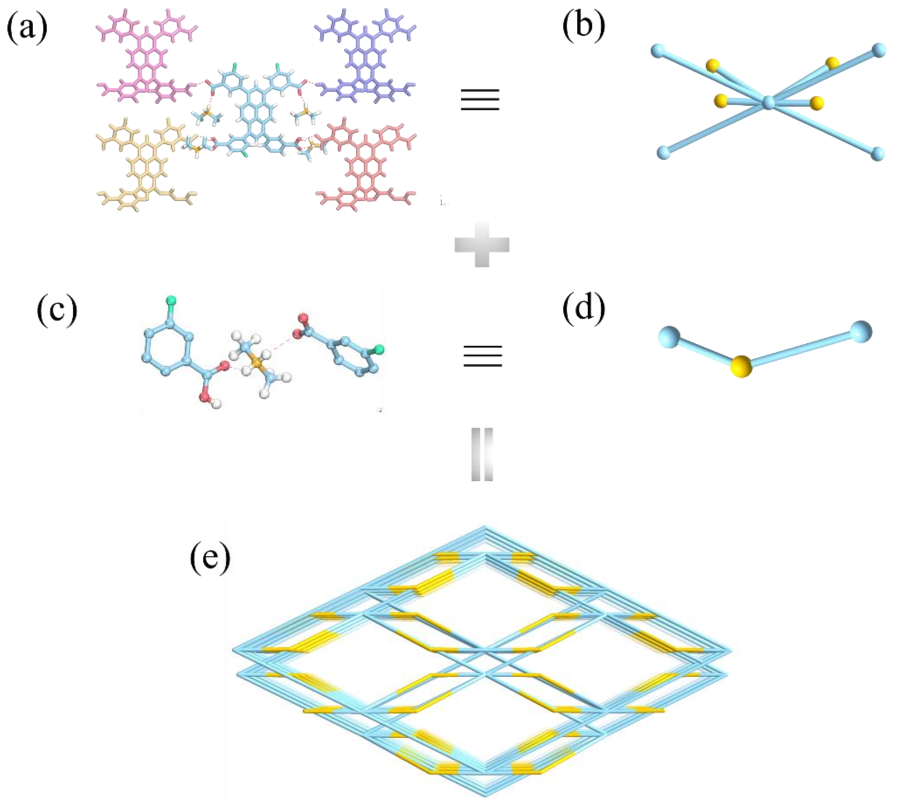

Figure S6. (a) Each $H_2PTTB\text{-}F^{2-}$ units in WYU-HOF-2 is connected to four adjacent $H_2PTTB\text{-}F^{2-}$ units and four adjacent $HDMA^+$ units; (b) 8-c node simplified from $H_2PTTB\text{-}F^{2-}$ unit; (c) each $HDMA^+$ units is bridged by two $H_2PTTB\text{-}F^{2-}$ units; (d) 2-c node simplified from $HDMA^+$ units; (d) the 2,8-connected topological net of WYU-HOF-2 with point symbol of $\{4^6{\cdot}6^{18}{\cdot}10^4\}\{4\}^2$.

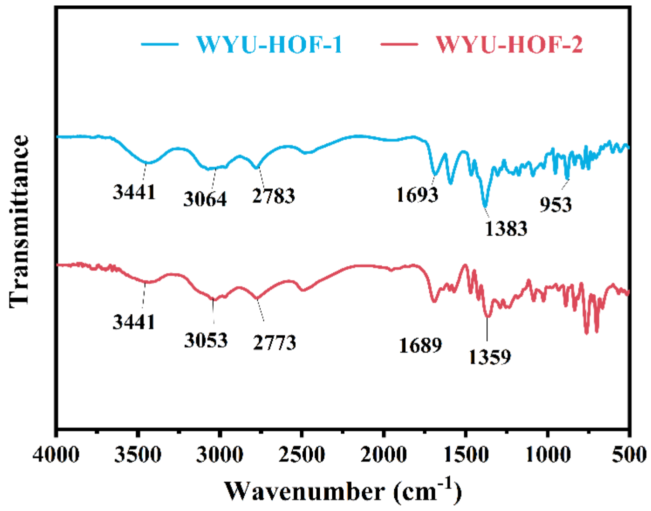

Figure S7. The FT-IR spectra of WYU-HOF-1 and WYU-HOF-2.

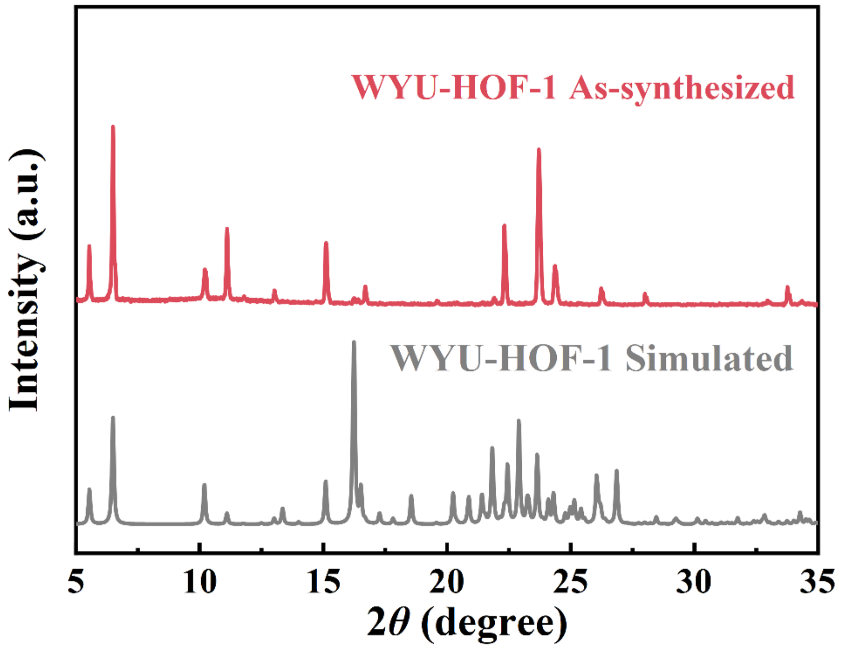

Figure S8. Experimental and simulated powder XRD patterns of WYU-HOF-1.

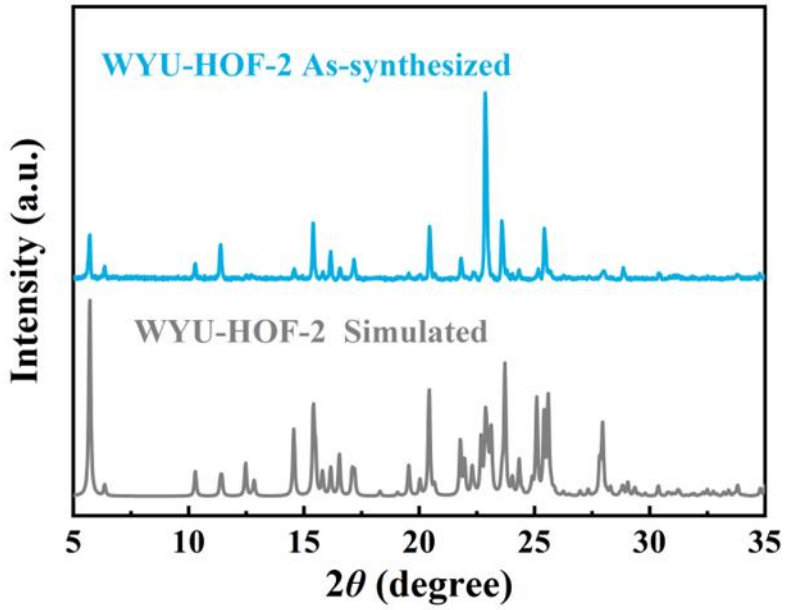

Figure S9. Experimental and simulated powder XRD patterns of WYU-HOF-2.

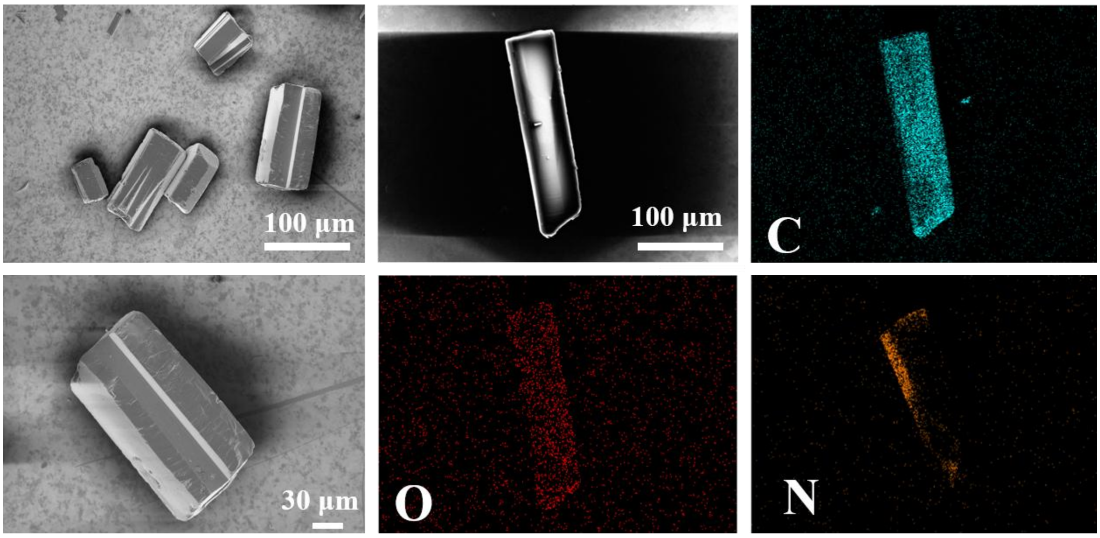

Figure S10. SEM and corresponding EDS mapping images of WYU-HOF-1.

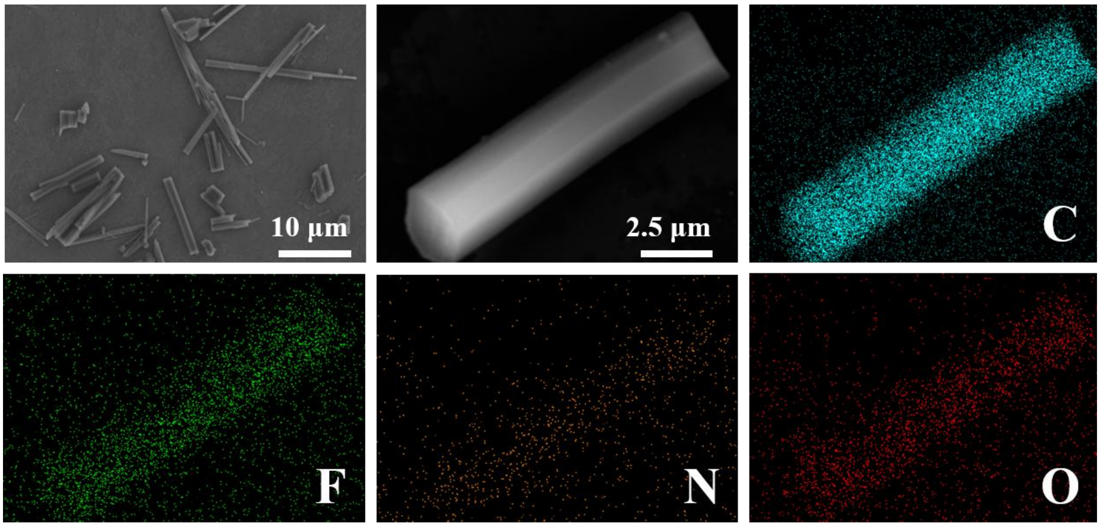

Figure S11. SEM and corresponding EDS mapping images of WYU-HOF-2.

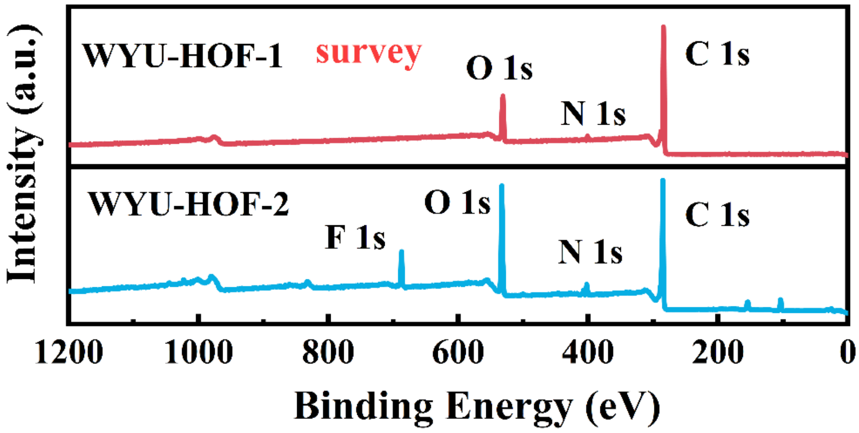

Figure S12. XPS survey of WYU-HOF-1 and WYU-HOF-2.

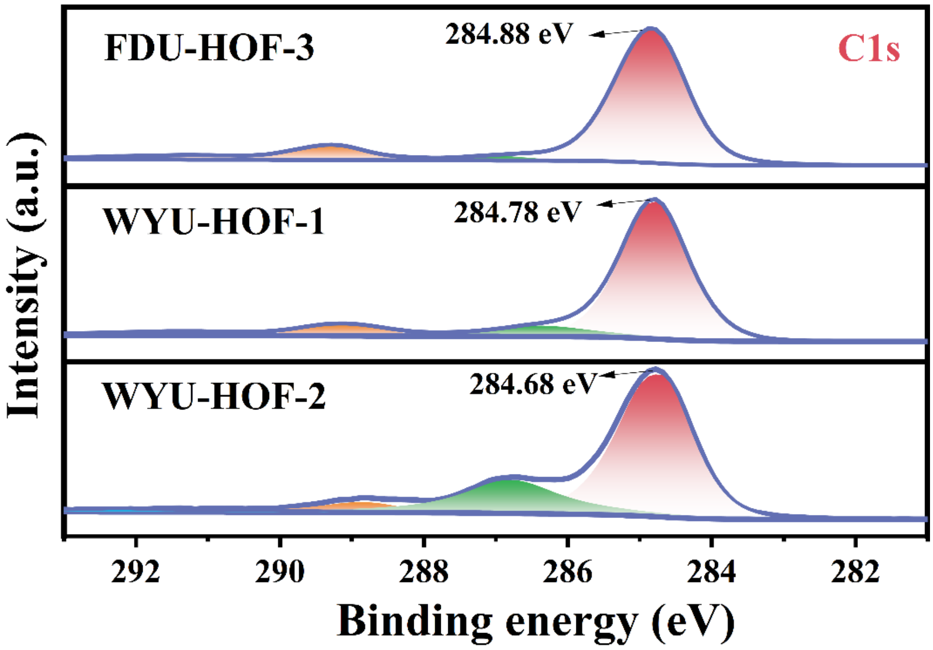

Figure S13. High-resolution XPS spectra of C 1s in FDU-HOF-3, WYU-HOF-1 and WYU-HOF-2.

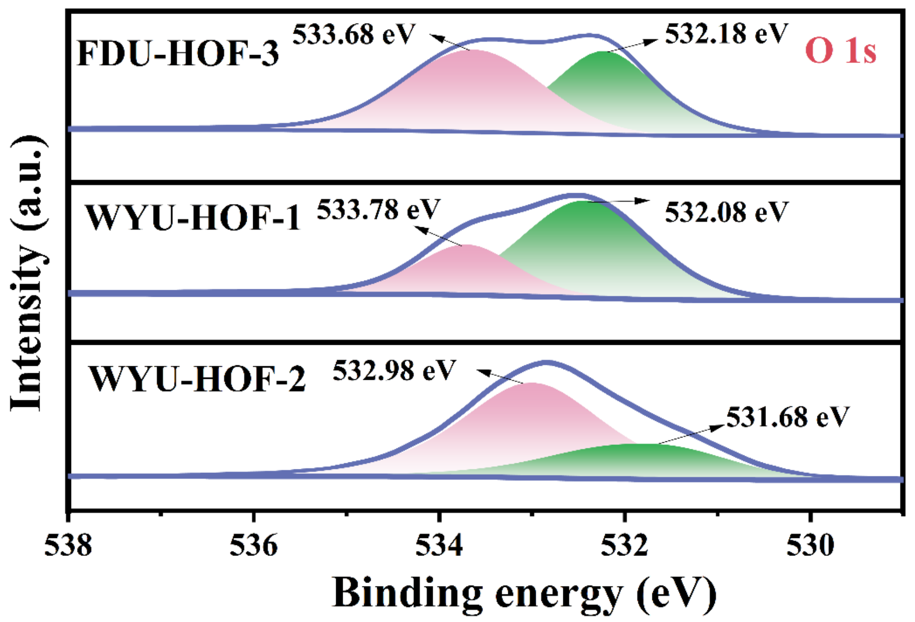

Figure S14. High-resolution XPS spectra of O 1S in FDU-HOF-3, WYU-HOF-1 and WYU-HOF-2.

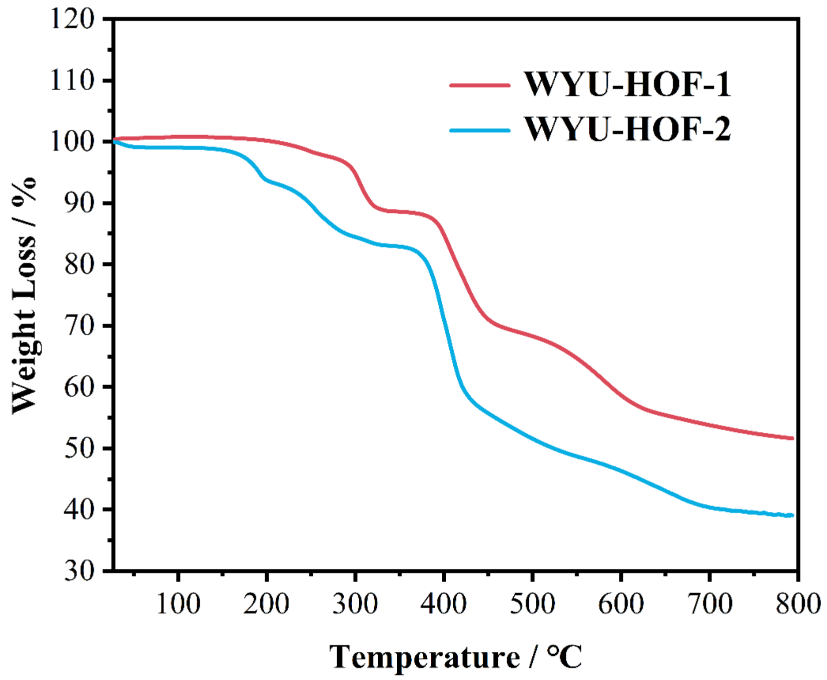

Figure S15. The TG curves of activated WYU-HOF-1 and WYU-HOF-2 samples.

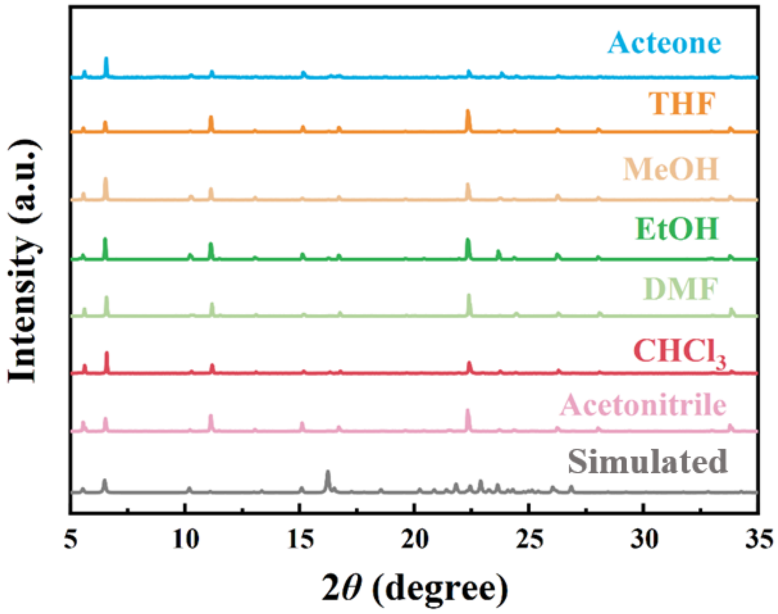

Figure S16. Stability test of WYU-HOF-1 in different solvents for 7 days.

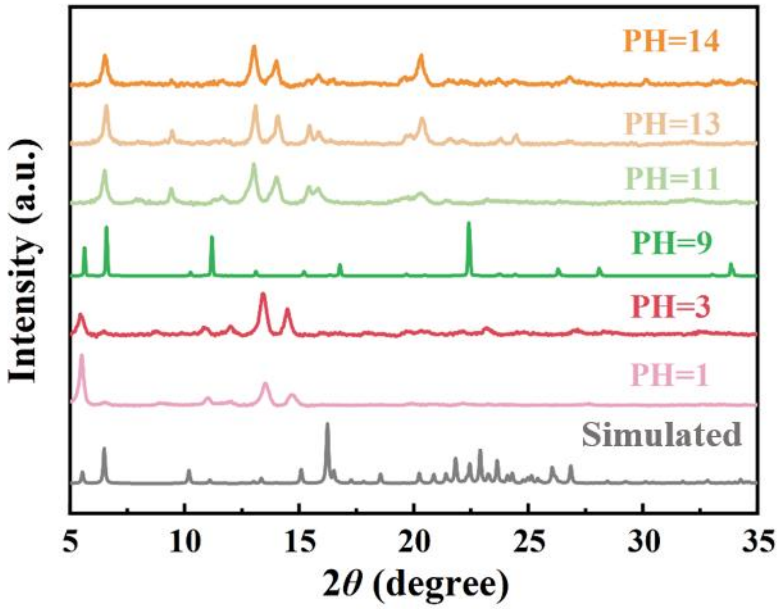

Figure S17. Stability test of WYU-HOF-1 in aqueous solutions with a pH range from 1 to 14 for 7 days.

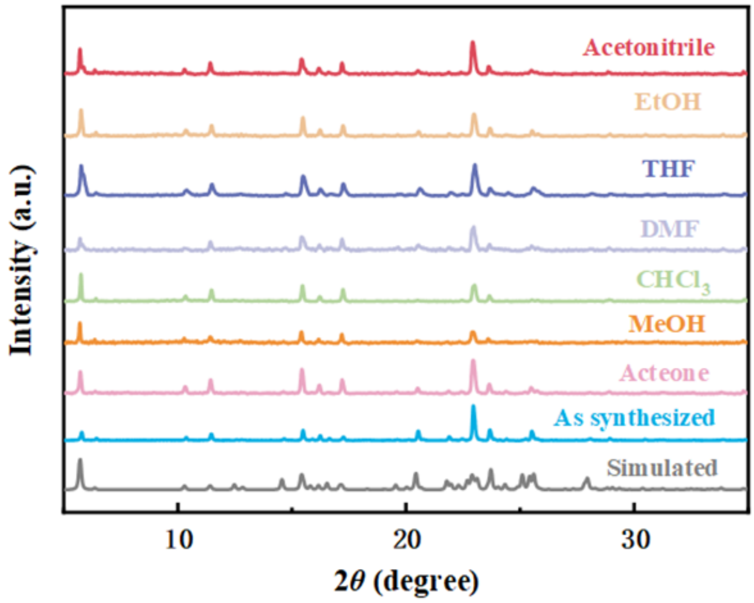

Figure S18. Stability test of WYU-HOF-2 in different solvents for 7 days.

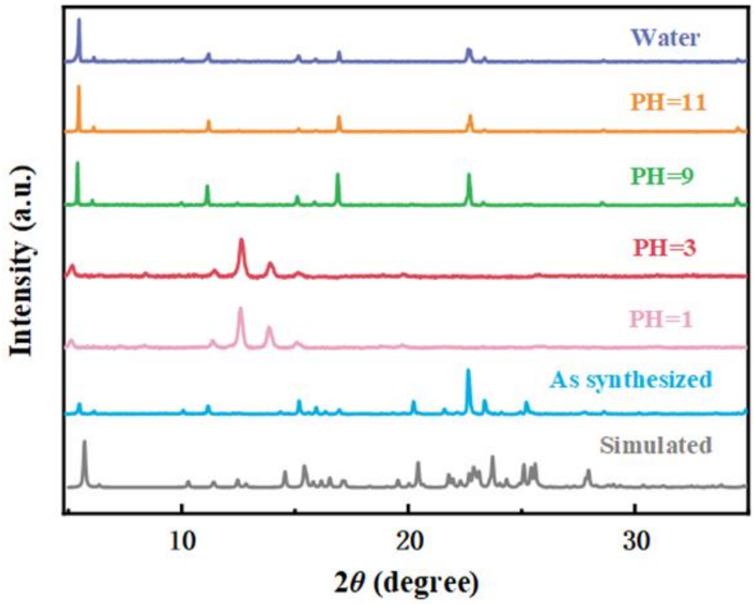

Figure S19. Stability test of WYU-HOF-2 in aqueous solutions with a pH range from 1 to 14 for 7 days.

5. Photoelectric Property

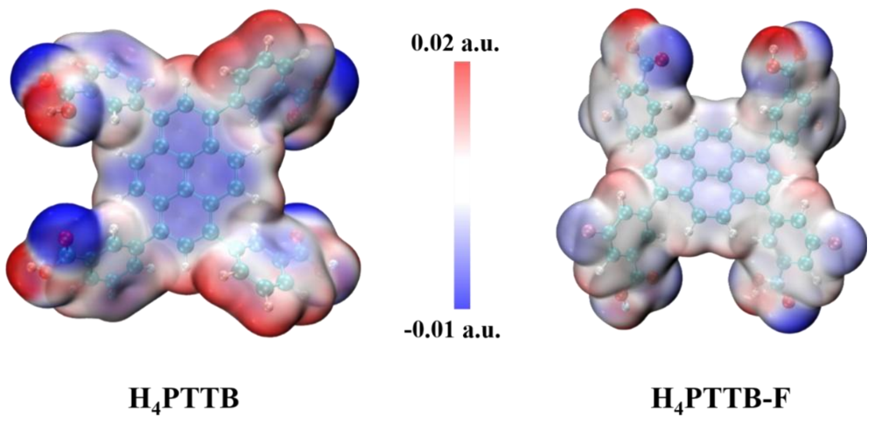

Figure S20. Electrostatic potential surface of H_4PTTB-H (left) and H_4PTTB-F (right).

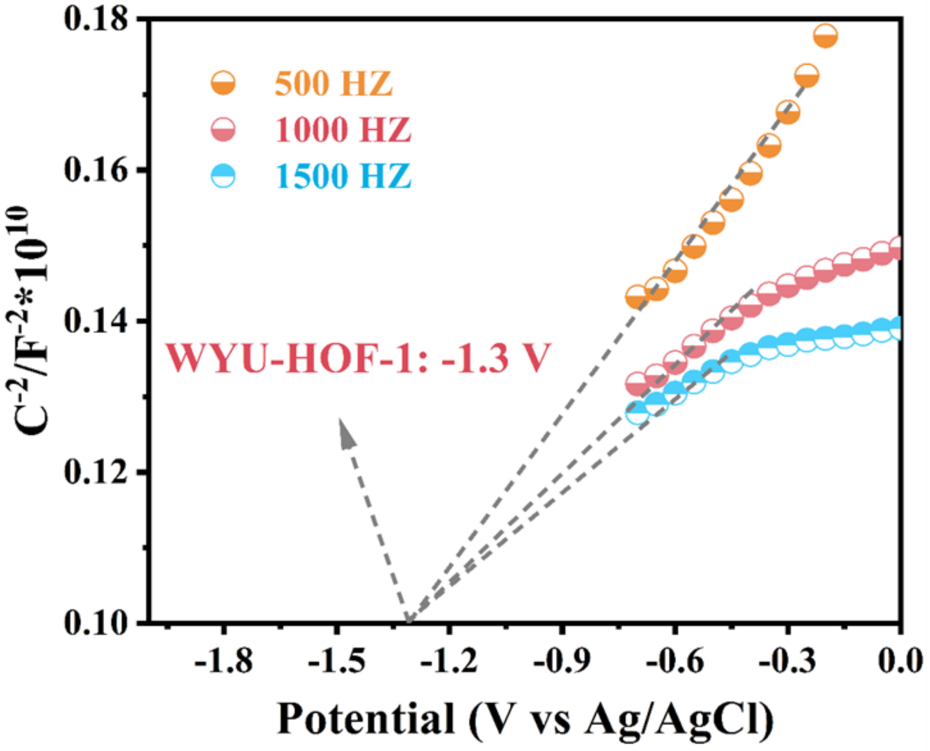

Figure S21. Mott–Schottky plot of WYU-HOF-1 in 0.1 M Na_2SO_4 aqueous solution (PH 6.5).

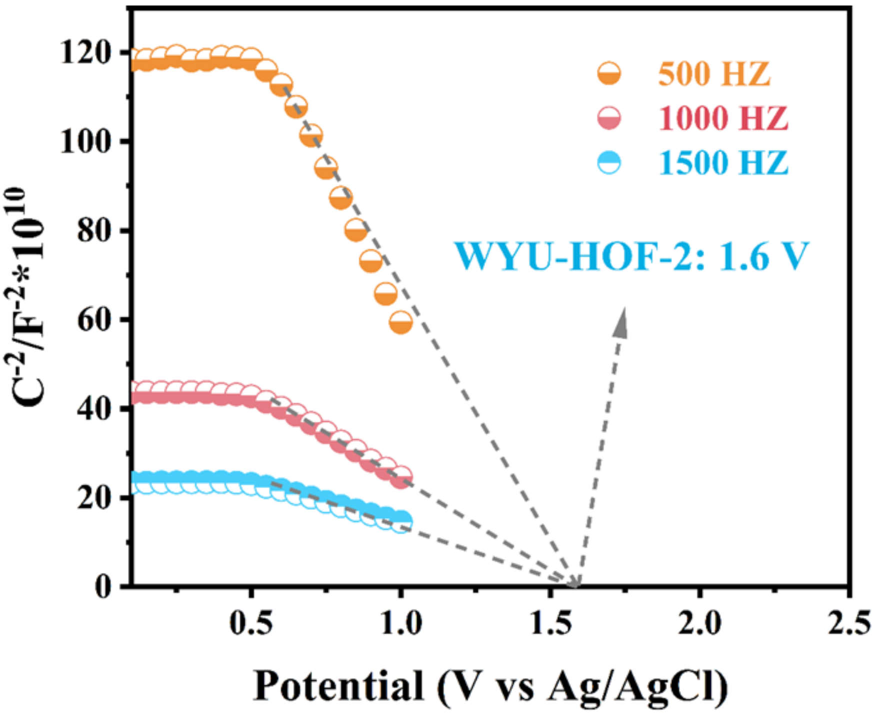

Figure S22. Mott–Schottky plot of WYU-HOF-2 in 0.1 M Na_2SO_4 aqueous solution (pH 6.5).

4.1. The equation for the conversion of Ag/AgCl relative to a normal hydrogen electrode (NHE)

The conduction band (CB) relative to the normal hydrogen electrode (NHE) at pH = 0 was obtained according to the following formula:

$E_{Ag/AgCl} = E_{RHE}-0.0591*pH-0.197$ (1)

$E_{NHE} = E_{RHE}-0.0591*pH$ (2)

The pH in equation (1) derived from the Na_2SO_4 solution is 6.5, while the pH in equation (2) is 0.

6. Photocatalytic H_2O_2 generation

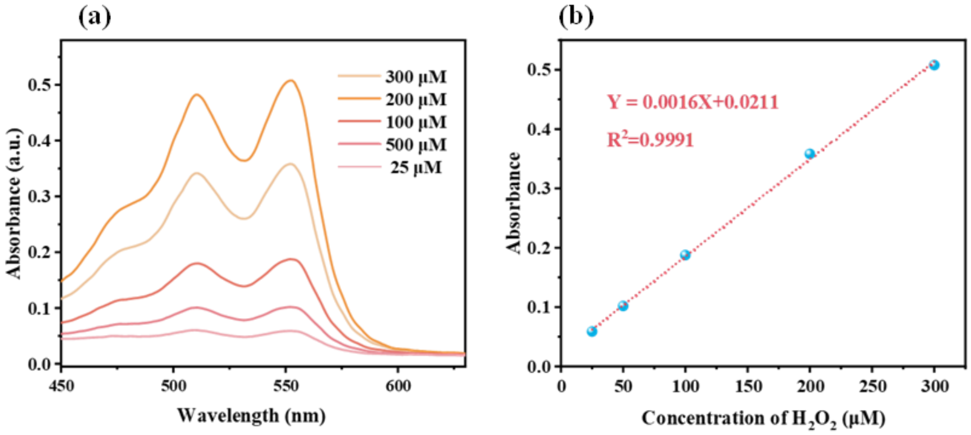

Figure S23. Standard curve for H_2O_2 detection. (a) UV-vis absorption spectra of DPD-POD obtained from H_2O_2 solutions with different concentrations. (b) corresponding calibration curve at the absorption peak of 551 nm.

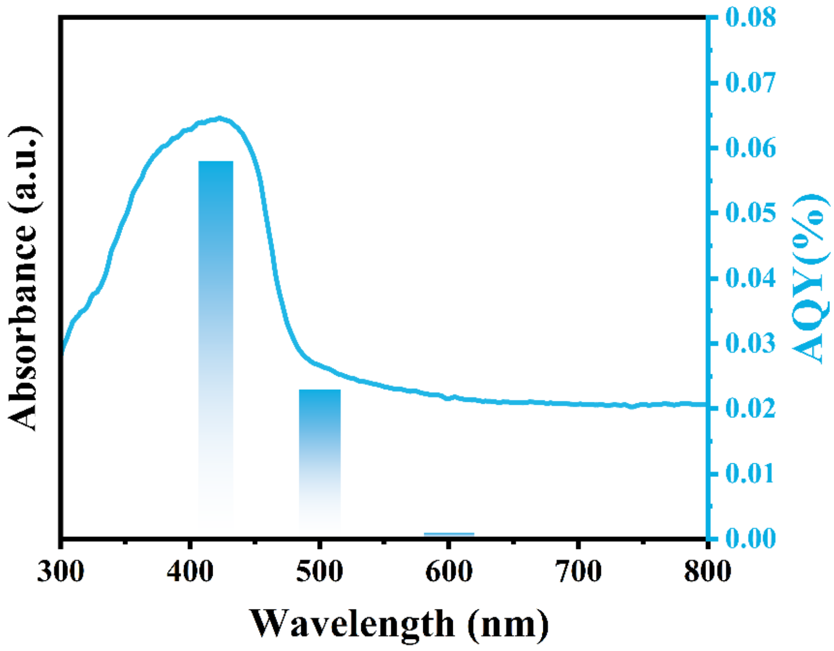

Figure S24. UV–vis diffuse reflection spectroscopy (UV/Vis DRS) spectrum and the apparent quantum yield (AQY) of WYU-HOF-2 for H_2O_2 generation.

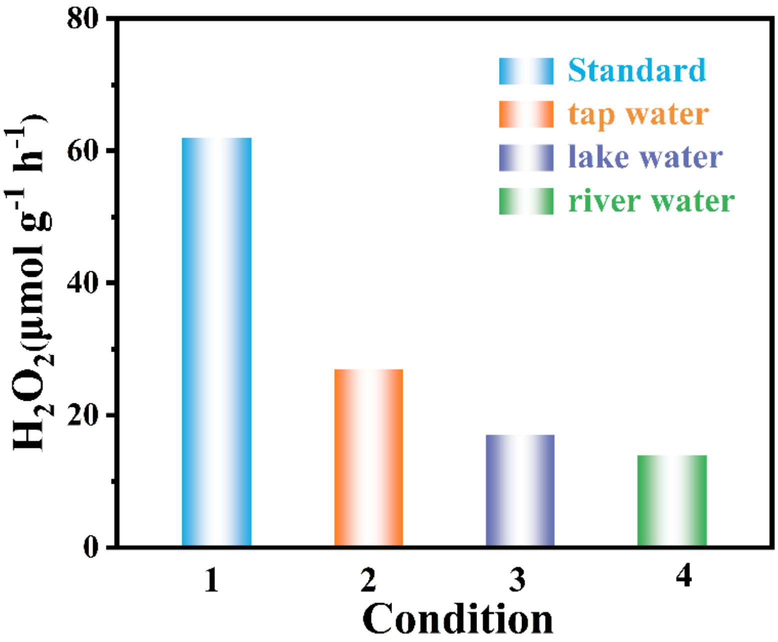

Figure S25. Photocatalytic H_2O_2 yield by WYU-HOF-2 in pure water, tap water, lake water and river water under visible-light irradiation.

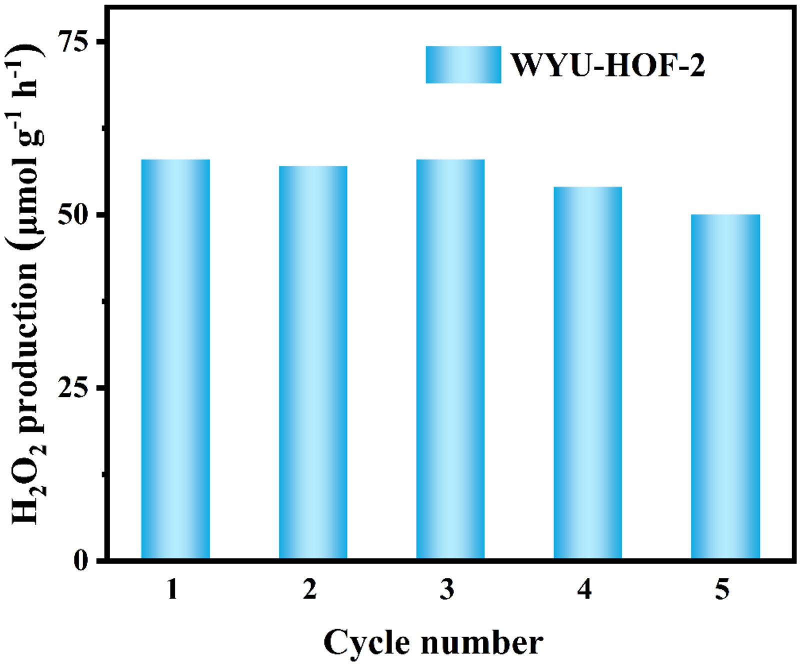

Figure S26. Recycle experiment

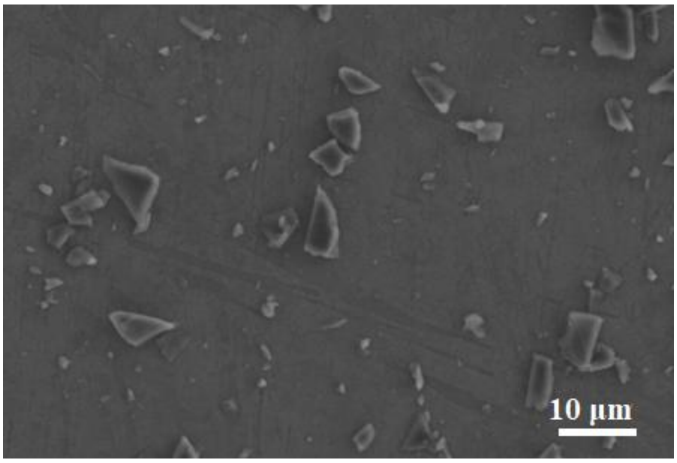

Figure S27. The SEM images of WYU-HOF-1 after photocatalytic reaction.

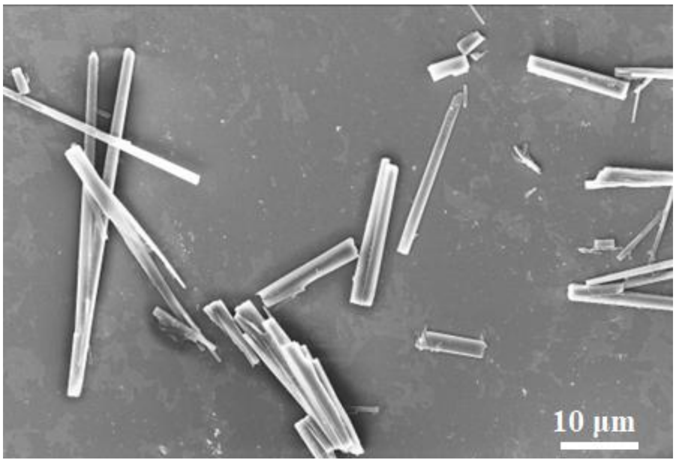

Figure S28. The SEM images of WYU-HOF-2 after photocatalytic reaction.

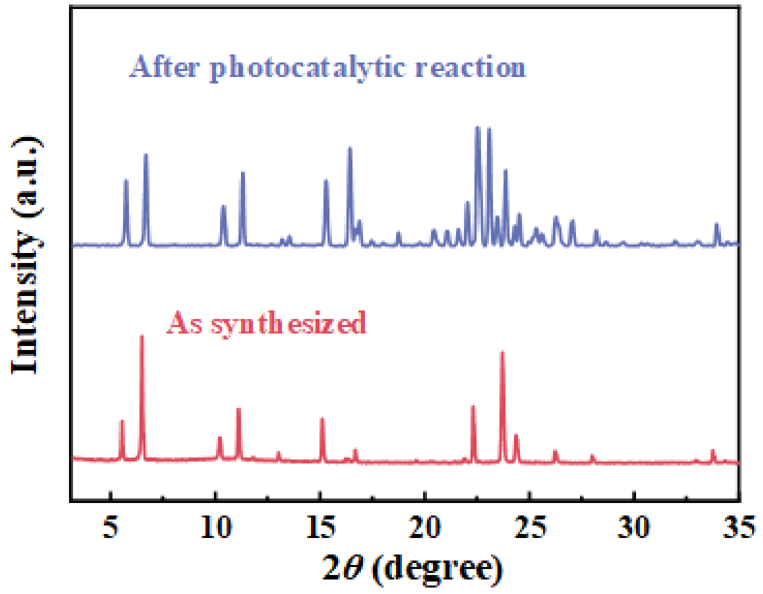

Figure S29. The PXRD patterns of WYU-HOF-1 before and after photocatalysis.

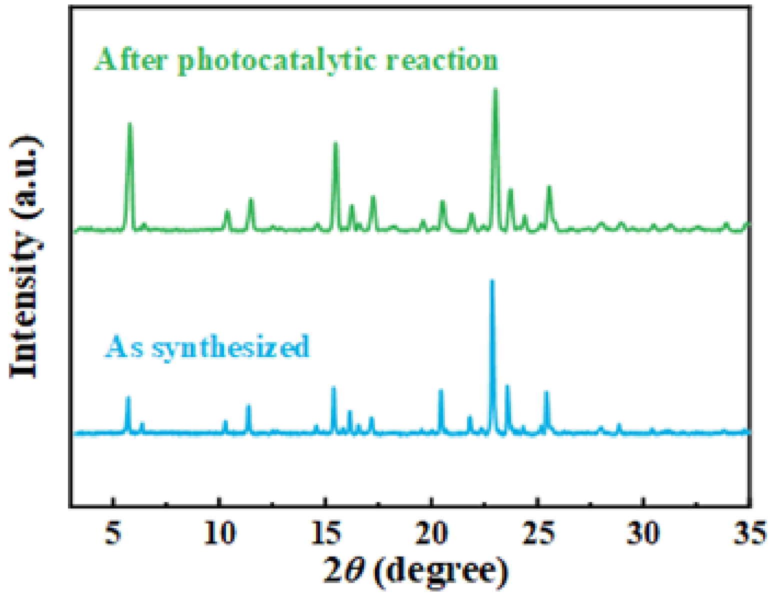

Figure S30. The PXRD patterns of WYU-HOF-2 before and after photocatalysis.

7. Reaction meachanism

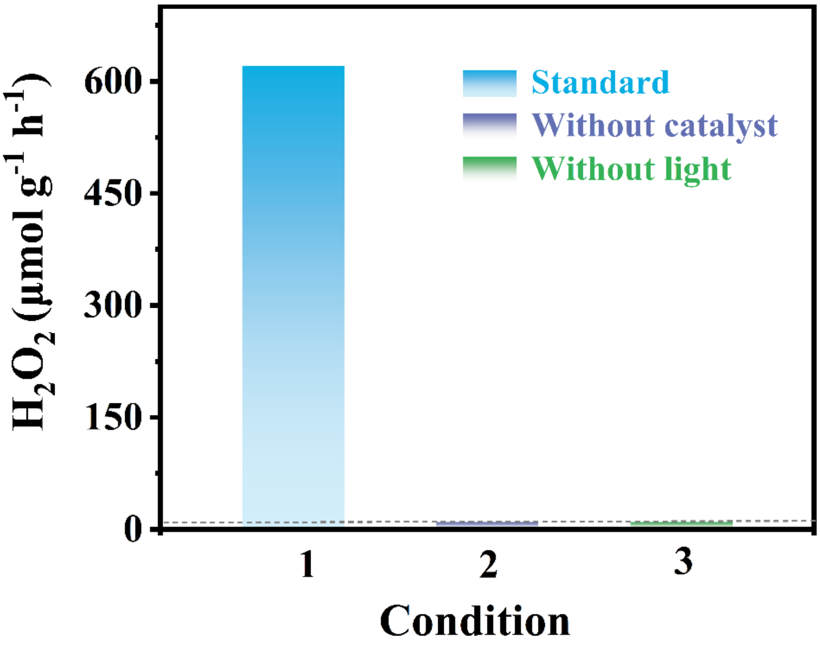

Figure S31. Controlled experiment of WYU-HOF-2 for H_2O_2 photosynthesis.

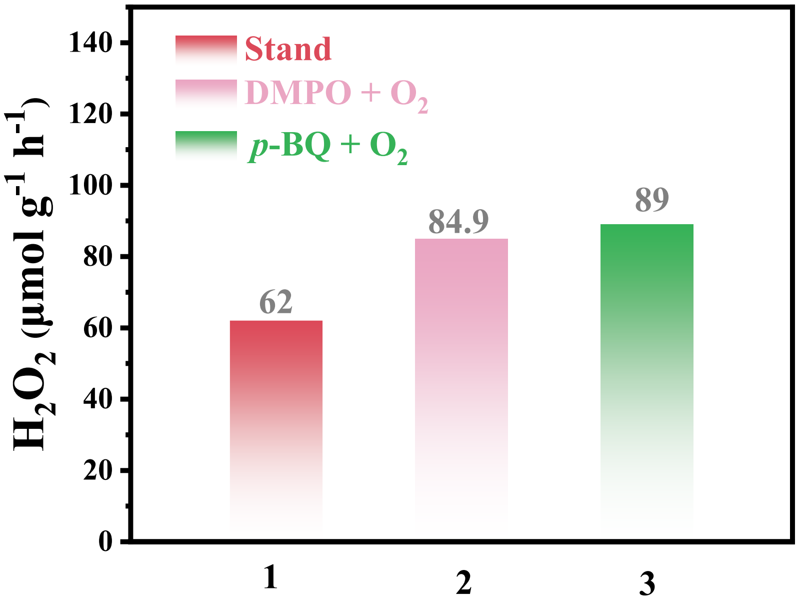

Figure S32. The amount of H_2O_2 generated over WYU-HOF-2 in the presence of different scavengers under irradiation for 1 h (p-BQ 5 mM; DMPO 4% V/V).

As shown in Figure S32, to avoid the potential influence of DMPO on the UV-vis measurements, another traditional $\bullet O_2^-$ scavenger (1,4-benzoquinone, p-BQ) was employed, and a similar enhancement phenomenon was observed. In the WYU-HOF-

2 system, the indirect single-electron pathway (via $\bullet O_2^-$) and the direct two-electron pathway coexist as two competing reaction channels, which collectively consume photogenerated electrons and oxygen under normal reaction conditions. When DMPO is added, it rapidly captures the superoxide radical ($\bullet O_2^-$), thereby suppressing the indirect single-electron pathway and redirecting the photogenerated electrons originally destined for this pathway to the direct two-electron pathway. Similar phenomenon and conclusion were also reported by Gu et al (*Angew. Chem. Int. Ed.* **2025**, *28,* e202508436). Due to the multiple charge transfer channels constructed in WYU-HOF-2, the direct two-electron pathway is kinetically more favorable and efficient. Therefore, when the reaction proceeds via a more efficient pathway, the overall yield of H_2O_2 is actually enhanced.

This phenomenon was not observed in the WYU-HOF-1 system, where the reaction is dominated by the indirect single-electron pathway. Unlike WYU-HOF-2, WYU-HOF-1 cannot sustain its yield through an equally efficient alternative pathway. Therefore, it exhibits the expected decrease in yield upon DMPO addition.

In summary, the increased H_2O_2 yield upon DMPO addition indicates the coexistence of dual pathways in WYU-HOF-2 and highlights the intrinsic superiority of the direct two-electron pathway.

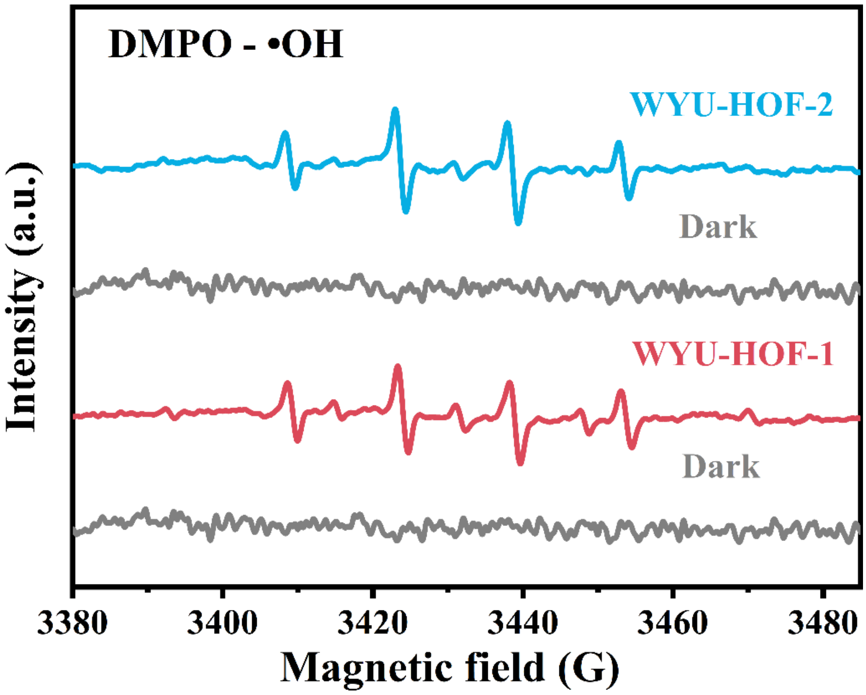

Figure S33. EPR spectra of DMPO-•OH for WYU-HOF-1 and WYU-HOF-2.

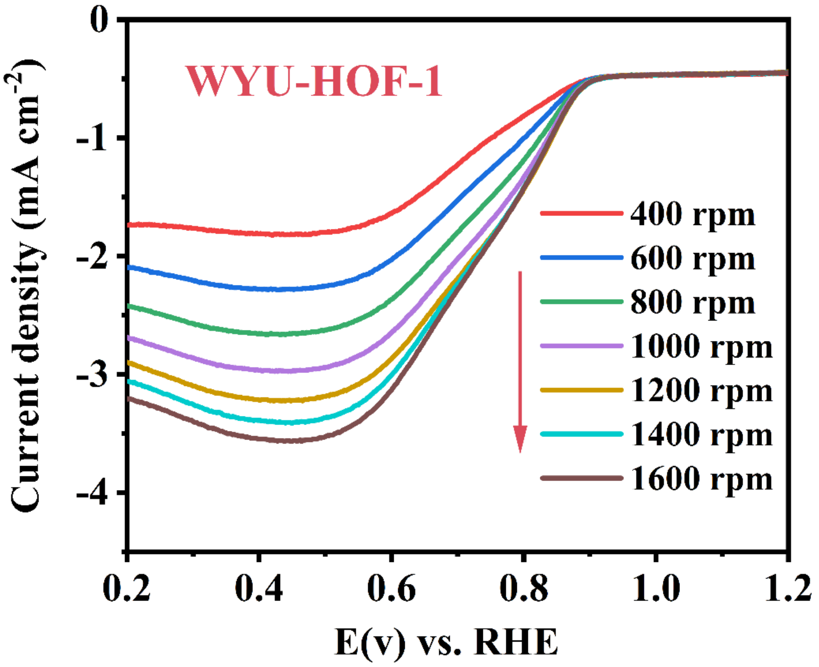

Figure S34. Linear-sweep RDE voltammograms of WYU-HOF-1 measured at different rotating speeds in phosphate buffer solution (pH = 7) with continuous O_2 purging.

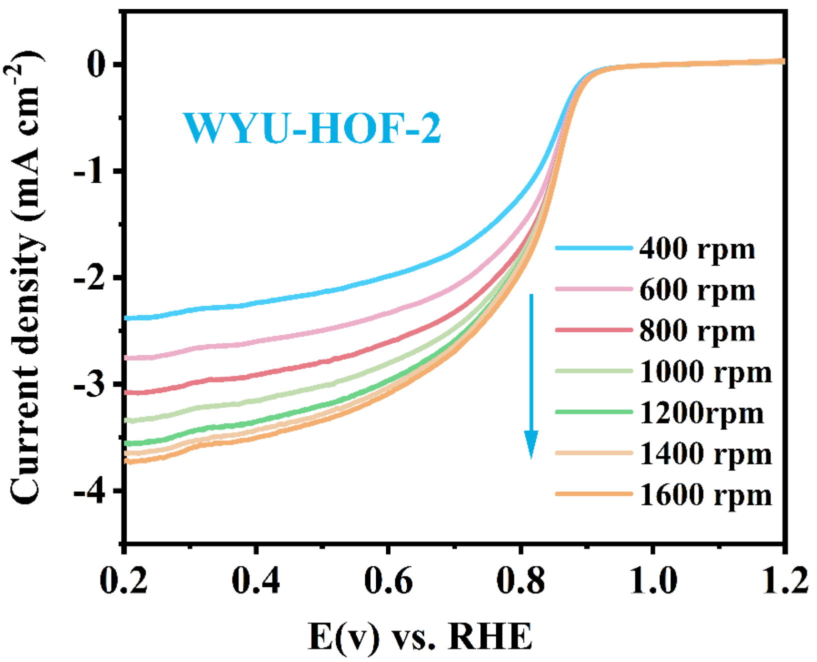

Figure S35. Linear-sweep RDE voltammograms of WYU-HOF-2 measured at different rotating speeds in phosphate buffer solution (pH = 7) with continuous O_2 purging.

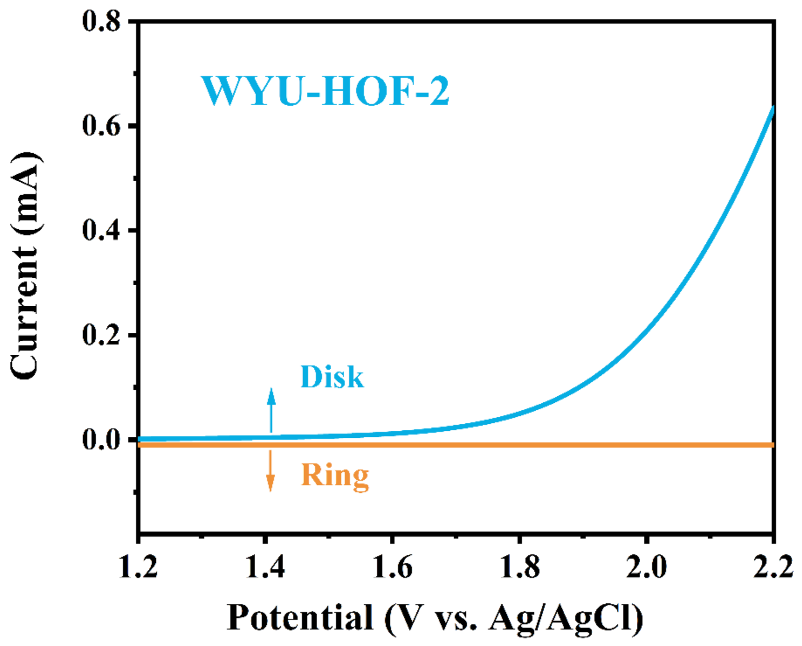

Figure S36. RRDE voltammograms of WYU-HOF-2 obtained in phosphate buffer (pH = 7) at rotation speed of 1600 rpm. The potential of Pt ring electrode was set at +0.23 V vs. Ag/AgCl to detect O_2.

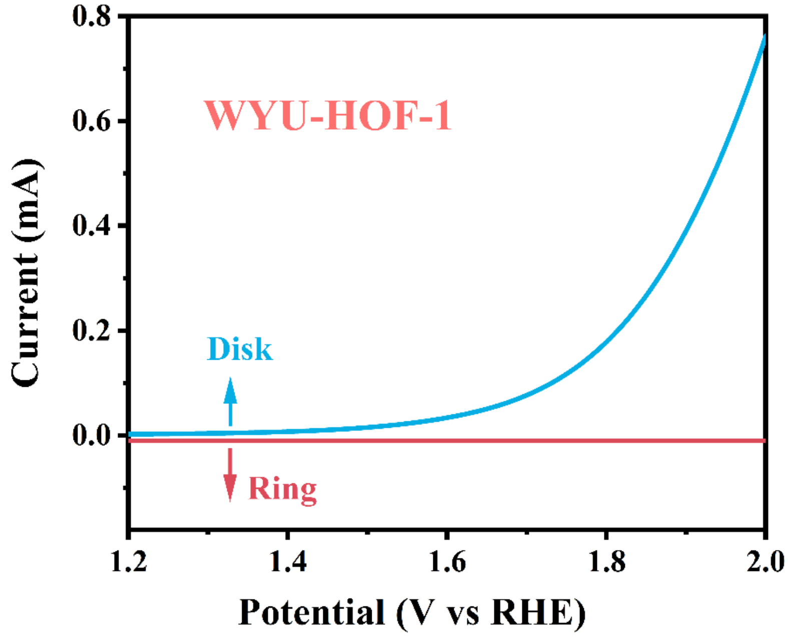

Figure S37. RRDE voltammograms of WYU-HOF-1 obtained in phosphate buffer (pH = 7) at rotation speed of 1600 rpm. The potential of Pt ring electrode was set at +0.23 V vs. Ag/AgCl to detect O_2.

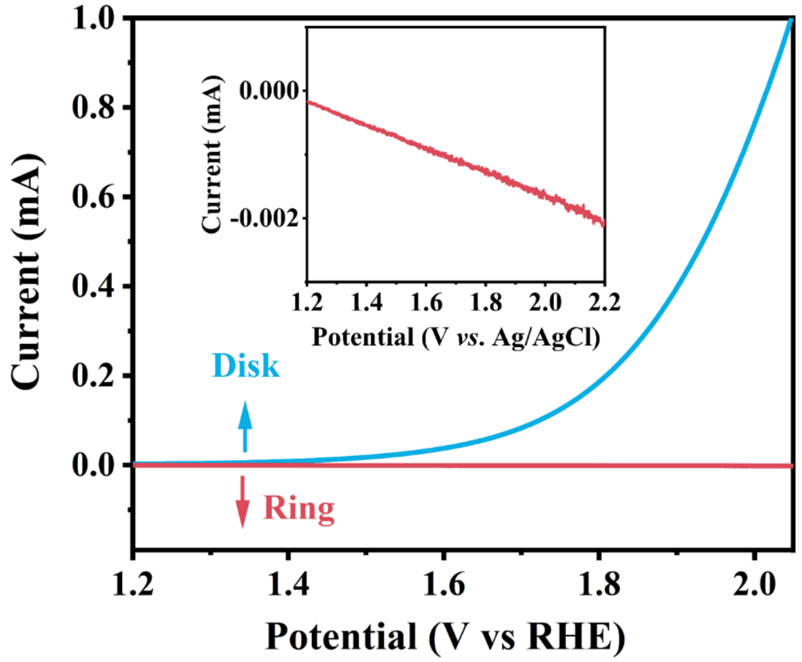

Figure S38. RRDE voltammograms of WYU-HOF-1 obtained in phosphate buffer (pH = 7) at rotation speed of 1600 rpm. The potential of Pt ring electrode was set at +0.6 V vs. Ag/AgCl to detect H_2O_2.

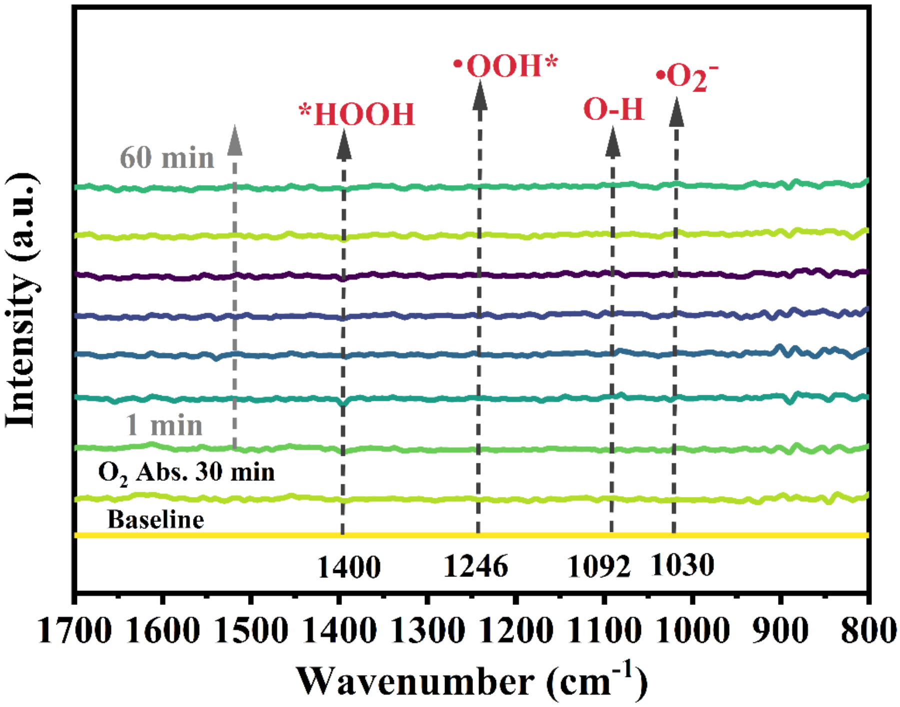

Figure S39. DRIFTS spectra of WYU-HOF-1 under the saturated O_2 condition in pure water.

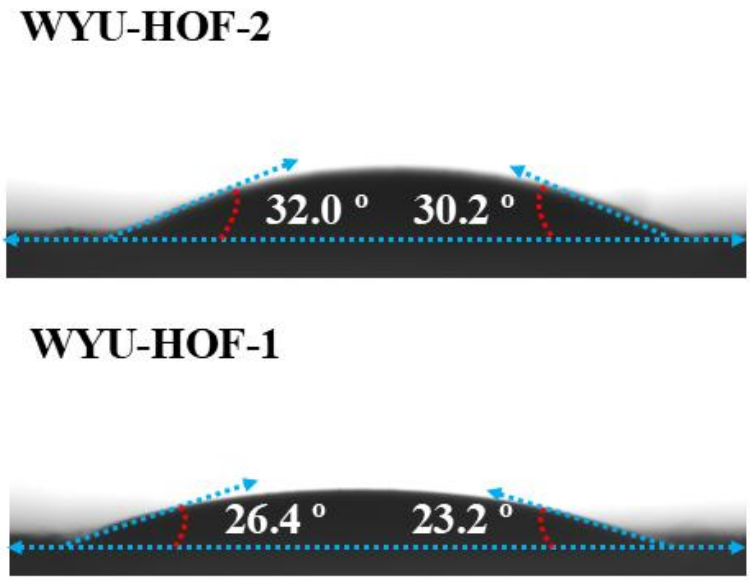

Figure S40. Water contact angle measurements for (a) WYU-HOF-1 and (b) WYU-HOF-2.

8. Theoretical calculation

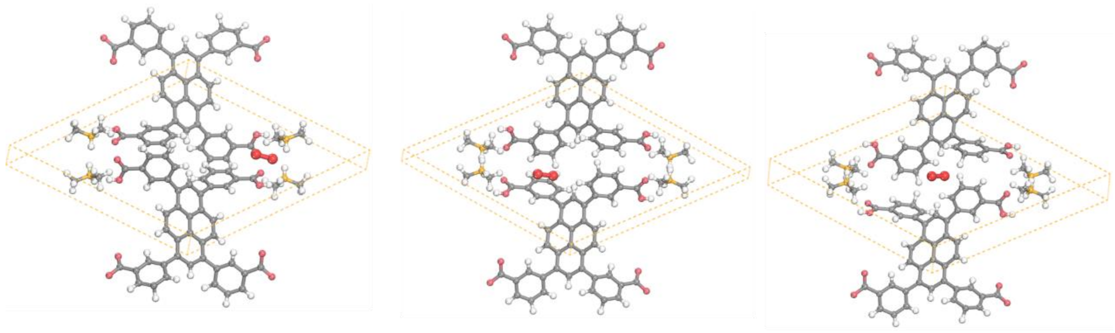

E_{ads} = 2.501 eV | E_{ads} = -0.428 eV | E_{ads} = 4.415 eV

Site A | **Site B** | **Site C**

Figure S41. Different O_2 adsorption configurations and adsorption energy for WYU-HOF-1.

Color codes: N, yellow; O, red; C, gray; H, white.

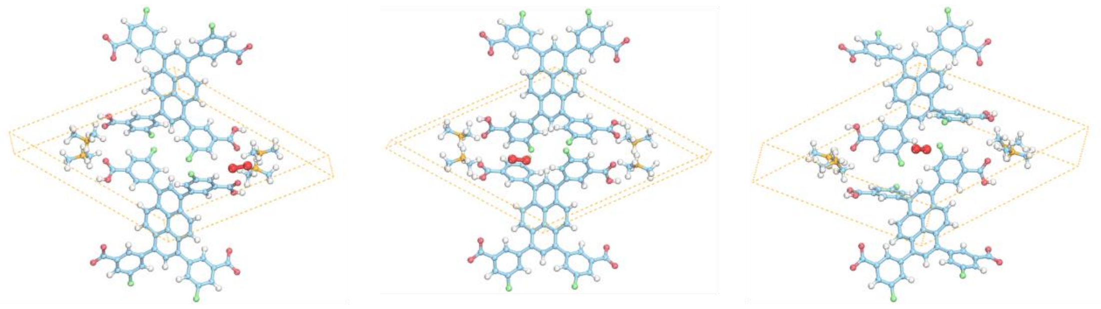

E_{ads} = 0.680 eV | E_{ads} = -1.997 eV | E_{ads} = -1.326 eV

Site A | **Site B** | **Site C**

Figure S42. Different O_2 adsorption configurations and adsorption energy for WYU-HOF-2.

Color codes: N, yellow; F, green; O, red; C, blue; H, white.

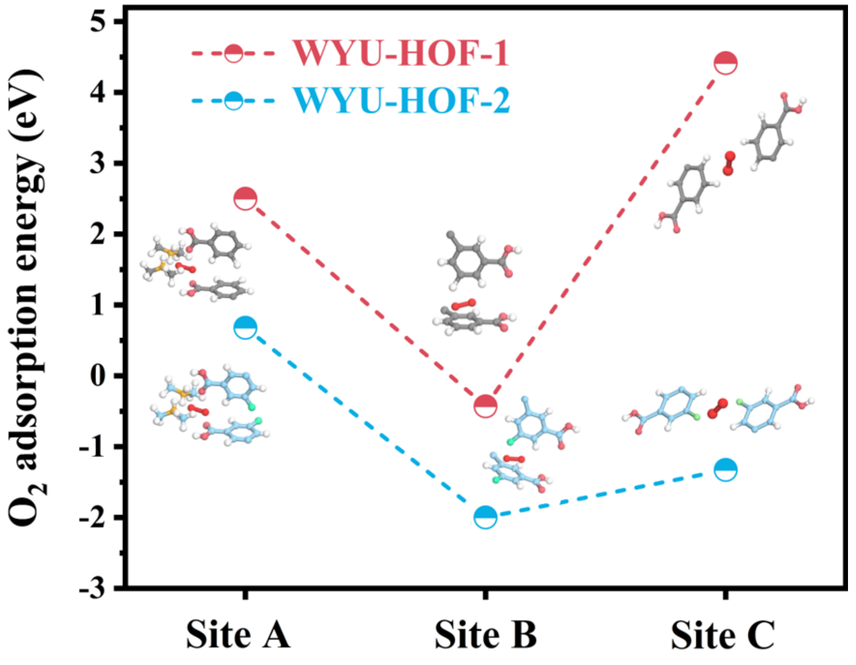

Figure S43. The adsorption energy of O_2 for WYU-HOF-1 and WYU-HOF-2.

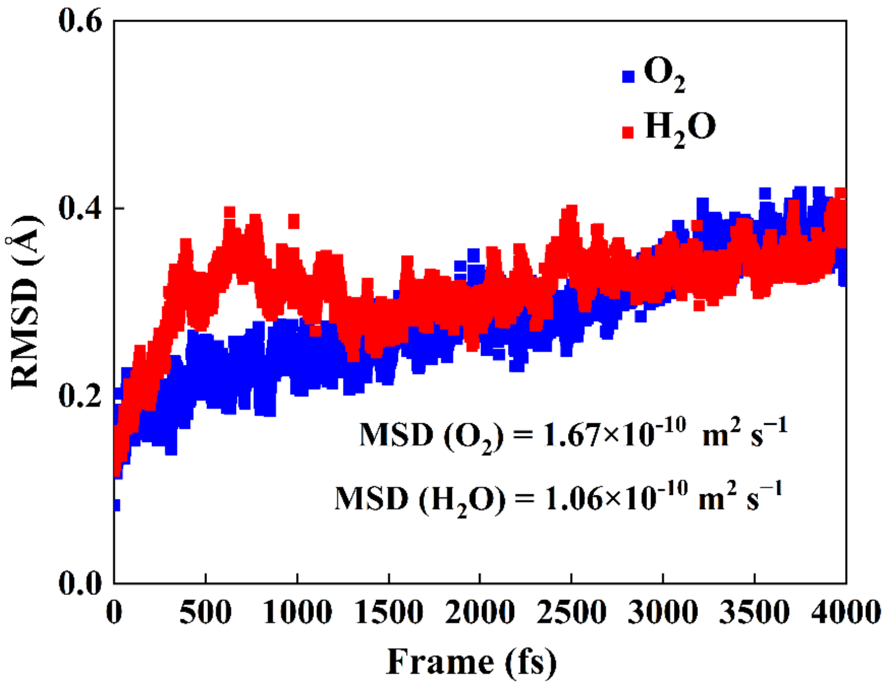

Figure S44. MSD of O_2 and H_2O diffusion in WYU-HOF-2.

NMR spectra of the Ligand

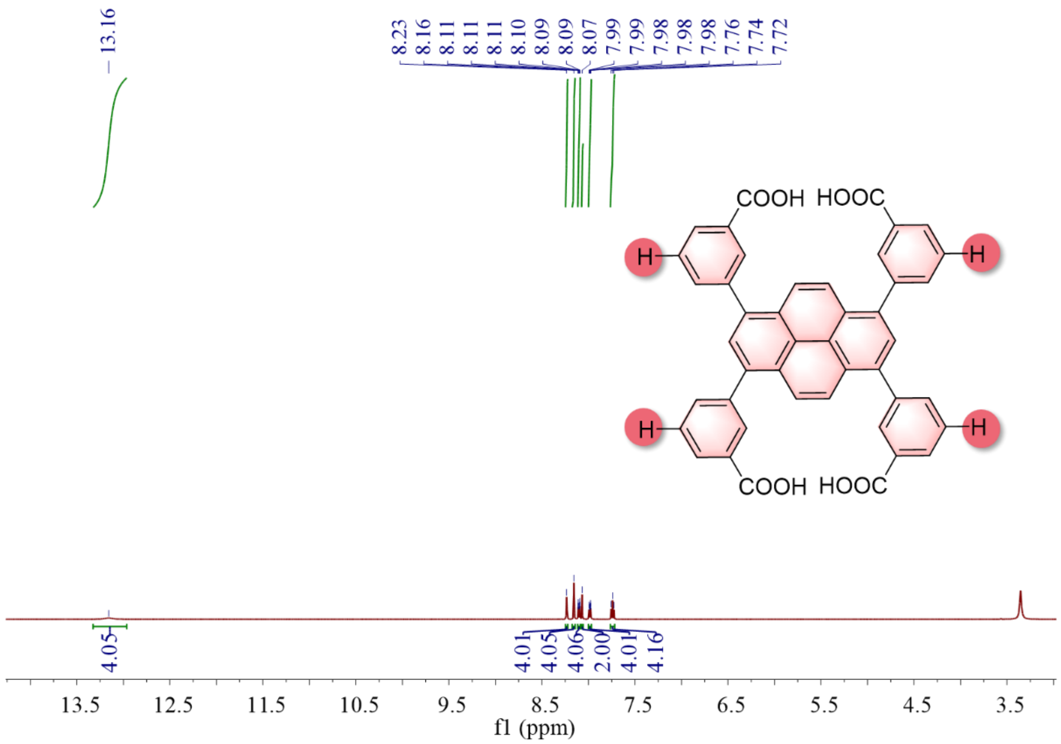

^{1}H NMR ((500 MHz, DMSO-d$_6$) spectrum of 1,3,6,8-tetrakis(3-carboxyphenyl) pyrene (**H$_4$PTTB**)

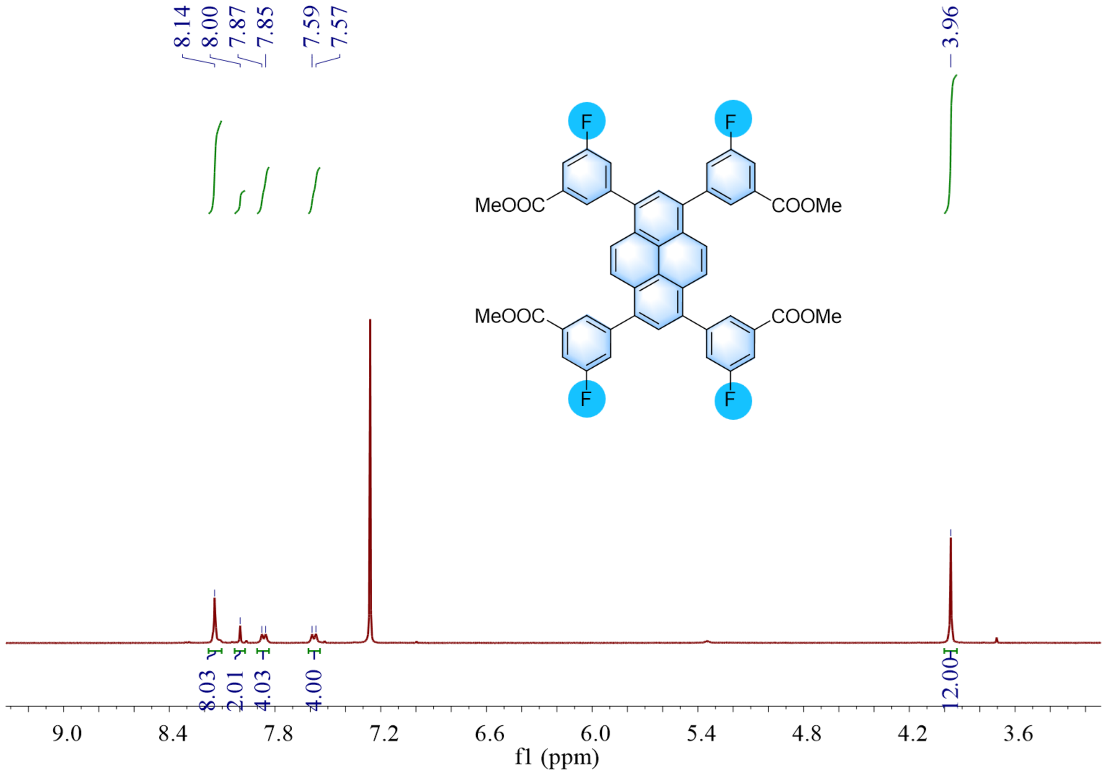

^{1}H NMR ((500 MHz, $CDCl_3$) spectrum of 1,3,6,8-tetrakis(3-fluoro-5-(methoxycarbonyl) phenyl) pyrene (**1b**)

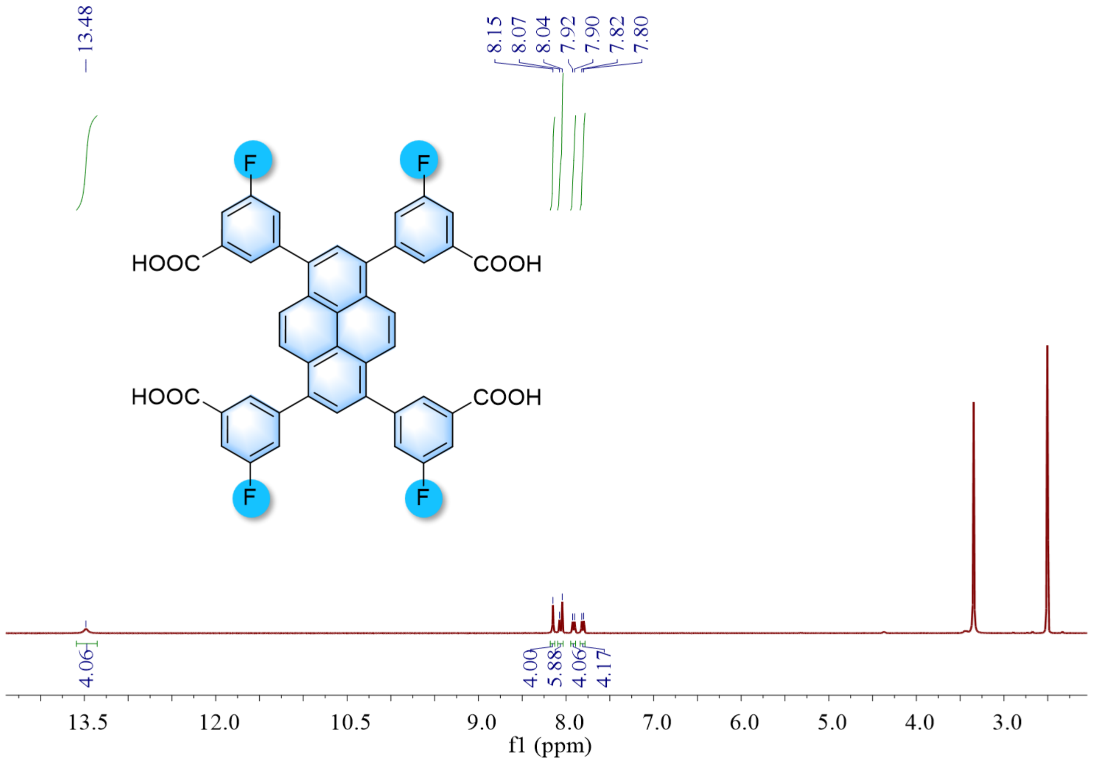

^{1}H NMR ((500 MHz, DMSO-d_6) spectrum of 1,3,6,8-tetrakis(3-fluoro-5-(carboxyphenyl)) pyrene

(**H_4PTTB-F**)

Reference

[1] Sluis, P. V. D.; Spek, A. L. BYPASS. an Effective Method for the Refinement of Crystal Structures Containing Disordered Solvent Regions. *Acta Crystallogr*. **1990**, *A46*, 194−201.

[2] (a) Li, Y.-X.; Hu, Y.; Bae, H.-S.; Du, J.; Zhao, S.; Pan, D.; Choi, W. Designing 1-nm-Thick MOF Nanosheets with Donor–Acceptor Complexes for Photosynthesis of H_2O_2 Using Water and Dioxygen Only. *ACS Nano* **2024**, *18*, 29233–29247; (b) Li, Y.; Guo, Y.; Fan, G.; Luan, D.; Gu, X.; Lou, X. W. Single Zn Atoms with Acetate-Anion-Enabled Asymmetric Coordination for Efficient H_2O_2 Photosynthesis. *Angew. Chem. Int. Ed.* **2024**, *63*, e202317572.

[3] (a) Zhao, X.; Qin, B.-B.; He, T.; Wang, H.-P.; Liu, J. Stable Pyrene-Based Metal−Organic Framework for Cyclization of Propargylic Amines with CO_2 and Detection of Antibiotics in Water. *Inorg. Chem*. **2023**, *62*, 18553−18562; (b) Zhao, X.; Zhao, Y.; Li, Y.-P.; Lyu, P.; Chen, C.; Mo, Z.-W.; Peng, C.; Liu, J.; Zhang, L. Engineering a pyrene MOF composite photocatalyst toward the formation of carbon dioxide radical anions through regulating the charge transfer from type II to Z scheme via a chemical bond-modulated strategy. *Inorg. Chem. Front*.,**2024**, *11*, 8489–8501; (c) Zhao, X.; Zhao, Y.; He, T.; Deng, J.-T.; Mo, Z.-W.; Liu, J. 2D pyrene-based metal–organic framework nanobelts as efficient photocatalysts for the coupling of thiols into disulfides. *Inorg. Chem. Front*., **2024**, *11*, 5700–5708.

[4] Song, X.; Wang, Y.; Wang, C.; Gao, X.; Zhou, Y.; Chen, B.; Li, P. Self-Healing Hydrogen-Bonded Organic Frameworks for Low-Concentration Ammonia Capture. *J. Am. Chem. Soc*. **2024**, *146*, 627–634.
